# Lytic reactions of drugs with lipid membranes†
†Electronic supplementary information (ESI) available: These might include comments relevant to but not central to the matter under discussion, limited experimental and spectral data, and crystallographic data. See DOI: 10.1039/c8sc04831b


**DOI:** 10.1039/c8sc04831b

**Published:** 2018-12-03

**Authors:** Hannah M. Britt, Clara A. García-Herrero, Paul W. Denny, Jackie A. Mosely, John M. Sanderson

**Affiliations:** a Chemistry Department , Durham University , South Road , Durham , DH1 3LE , UK . Email: j.m.sanderson@durham.ac.uk; b Department of Biosciences , Durham University , Stockton Road , Durham , DH1 3LE , UK

## Abstract

Propranolol is shown to undergo lipidation reactions in three types of lipid membrane: (1) synthetic single-component glycerophospholipid liposomes; (2) liposomes formed from complex lipid mixtures extracted from *E. coli* or liver cells; and (3) *in cellulo* in Hep G2 cells. Fourteen different lipidated propranolol homologues were identified in extracts from Hep G2 cells cultured in a medium supplemented with propranolol. This isolation of lipidated drug molecules from liver cells demonstrates a new drug reactivity in living systems. Acyl transfer from lipids to the alcoholic group of propranolol was favoured over transfer to the secondary amine. Migration of acyl groups from the alcohol to the amine was diminished. Other drugs that were examined did not form detectable levels of lipidation products, but many of these drugs did affect the lysolipid levels in model membranes. The propensity for a compound to induce lysolipid formation in a model system was found to be a predictor for phospholipidosis activity *in cellulo*.

## Abstract 2

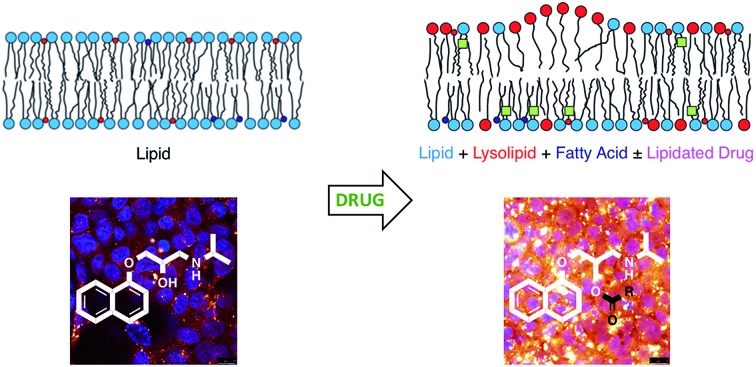
The involvement of drugs in direct chemical reactions with lipids may be linked to toxic effects in liver cell lines.

## Introduction

**Scheme 1:**
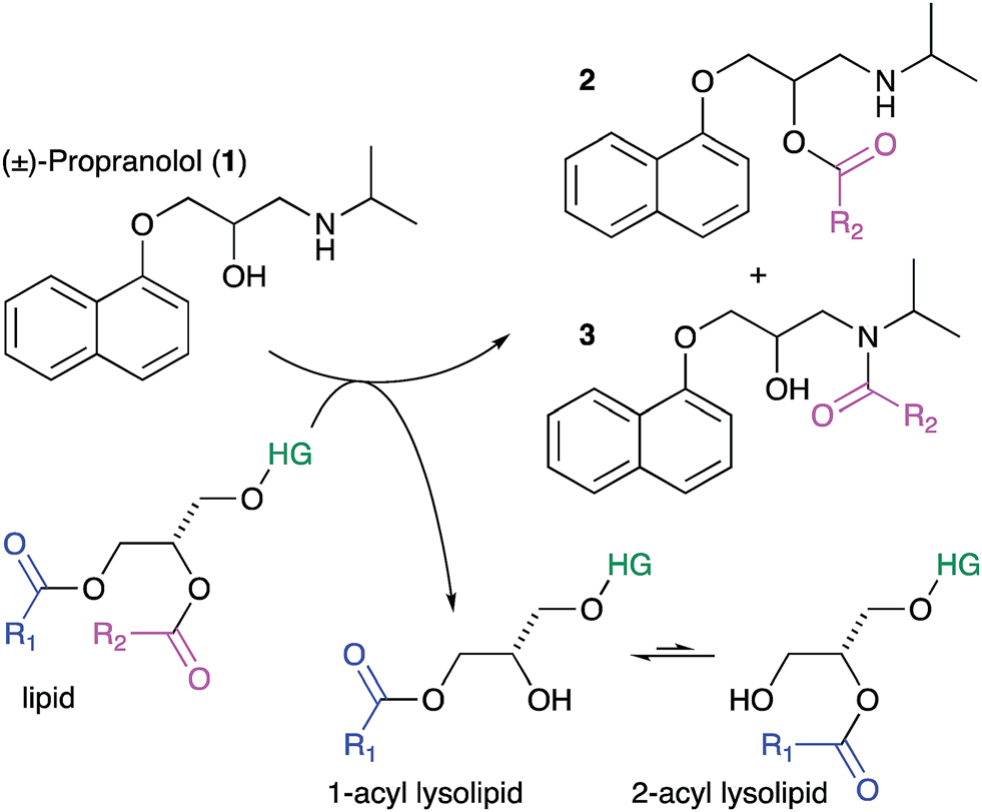
Lipidation reactions involving propranolol. Key: HG, headgroup.

Lipids are the key component of many materials, including liposomes used in technology applications and the membranes of cells and organelles in biological systems. In this article we examine whether organic molecules that partition into membranes, such as propranolol (**1**), can undergo direct chemical reactions with lipids (Scheme 1). Demonstration of such reactivity between membrane lipids and membrane-associated drugs *in vivo* would constitute a significant addition to our understanding of membrane chemistry. Reactivity of this nature is likely to lead to biological effects that may account for the adverse activities of some drugs and the unusual pharmacokinetic profiles of others. By contrast, exploiting this reactivity could offer opportunities for the purposeful design of new membrane-active compounds. The direct transfer of acyl groups from membrane lipids to suitably disposed molecules embedded within the membrane has a precedent, having been demonstrated for peptides *in vitro*.^1^–^3^ In these cases, peptide lipidation results from the high affinity of the peptide for the membrane, combined with the appropriate positioning of reactive groups in the membrane interface. These aminolysis reactions involve nucleophilic attack on a lipid ester group by a suitably disposed nucleophile on the peptide, typically a lysine ε-amino group or the N-terminal amino group, and lead to the formation of a lysolipid alongside the lipidated peptide.^1^ When the nucleophile is a serine hydroxyl group, the process is formally a transesterification. More recently, evidence has emerged to suggest that some membrane proteins may also be lipidated by direct transfer from the membrane. For example, the lipidation profile of the lens protein aquaporin-0 has been found to be highly complex.^4^ Aquaporin-0 has two lipidation sites that do not correspond to known consensus sequences for lipidation enzymes and are partially lipidated with numerous different acyl groups, with typically up to eight identifiable. The relative abundance of the acyl modifications at each site correlates with the acyl ester content of the lipids in the plasma membrane leaflet proximal to the site. Whilst these observations are not direct proof that this protein is lipidated by transfer from the membrane, they do give a strong indication that acyl transfer from the membrane is likely to be significant *in vivo*. Fundamentally, aquaporin-0 is a moderately high molecular weight protein that is permanently membrane-embedded *in vivo*, whilst peptides such as melittin that are lipidated *in vitro* are of medium molecular weight with modest membrane affinity. It is therefore of key interest to establish whether low molecular weight organic molecules, with concomitantly lower membrane affinity, are able to undergo the same process. We selected the β-blocker propranolol to probe this reactivity as it has both alcoholic and amine functionalities alongside well-characterised membrane binding characteristics.^5^–^7^ Furthermore, synthetic versions of *O*- and *N*-acyl propranolol derivatives have been described as prodrugs,^8^–^11^ providing precedents for understanding their properties *in vitro* and synthesising reference compounds for this work. In addition, propranolol is known to induce phospholipidosis,^12^,^13^ an adverse activity associated with disorders in lysosomal phospholipid storage, characterised by the formation of lamellar bodies visible by microscopy of susceptible cells.^14^–^20^ In principle therefore, propranolol may undergo both aminolysis and transesterification reactions, both of which lead to the formation of lysolipids, as shown in Scheme 1 for reaction with the acyl group at the sn-1 position of the lipid (although reaction can occur at either the sn-1 or sn-2 position). The lysolipid product would be formed as an equilibrium mixture of the 1- and 2-acyl species.^1^ All of the products could be reasonably expected to display significant biological activity as a consequence of the presence of a fatty acyl chain. Lysolipids in particular, are capable of inducing significant deleterious biological effects at levels as low as 1 mol%.^21^

Lysolipids can also be formed by lipid hydrolysis, with concomitant formation of a fatty acid. Membrane-associated drugs have the potential to influence the rate of this hydrolysis by either changing the bulk properties of the membrane, mediated by secondary effects on interfacial water activity, or by direct involvement in acid or base catalysis.^22^,^23^ This article describes the propensity of a number of membrane-active drugs to undergo direct lipidation reactions with membrane lipids, or promote other lytic reactions of lipids.

## Experimental

### Materials

Phospholipids and lysolipids, including *E. coli* Extract Polar (catalogue number 100600P) and Liver Polar Lipid Extract (Bovine, catalogue number 181108P) were purchased as powders from Avanti Polar Lipids (*via* Instruchemie B.V., The Netherlands). Propranolol was used as a racemic mixture.

### Liposome preparation and sample set up

Liposomes were prepared by extrusion of lipid dispersions 10× through polycarbonate filters (Whatman) with 100 nm track-etched pores, using a LIPEX thermobarrel extruder (Northern Lipids Inc., BC, Canada). All experiments were conducted at concentrations of 1.27 mM for lipids and (when present) 0.127 mM for membrane binding compounds. Using published partitioning data^5^ these concentrations produce a bound propranolol to lipid ratio of about 1 : 30. Samples at pH 7.4 were buffered using bicarbonate at a NaCl concentration of 90 mM. Calibration curves for analyte concentration were generated by fitting a logistic model to data obtained using authentic standards of lysolipids, propranolol and acyl propranolol derivatives at known concentrations.

### Hep G2 culture and extraction

Hep G2 cells from ATCC were grown to confluence at 37 °C, 5% CO_2_, and 95% humidity in Dulbecco's Modified Eagle Medium (DMEM) with 10% foetal bovine serum (Gibco/ThermoFisher). Cells (10^6^) were incubated at 37 °C, 5% CO_2_, and 95% humidity overnight to adhere. The medium was removed and replaced with either fresh medium (5 mL) for two controls, or medium containing 30 μM propranolol (5 mL). Following incubation for 72 h, the medium was removed from flasks and replaced with phosphate buffered saline (5 mL). Cells were collected by centrifugation for 10 min at 1000 × *g*, and decanted into a glass tube before extraction with CHCl_3_ : MeOH (2 : 1; 3 mL). The CHCl_3_ : MeOH solution was washed with H_2_O (0.6 mL), isolated, and the solvent removed *in vacuo*. Samples were resuspended in IPA : MeCN : H_2_O for liquid chromatography-mass spectrometry (LC-MS) analysis.

### Mass spectrometry

LC-MS was conducted on a Synapt G2-S (Waters Corp., UK) using electrospray ionisation. LC was conducted using an Acquity UPLC with a CSH C18 1.7 μm (2.1 × 150 mm) column with solvent A = H_2_O : MeCN 4 : 6 and B = MeCN : iPrOH, 1 : 9 (both A and B containing 10 mM NH_4_HCO_2_ + 0.1% formic acid) and the following A : B gradient: 60 : 40 to 57 : 43 over 2 min, 57 : 43 to 50 : 50 over 0.1 min, 50 : 50 to 46 : 54 over 9.9 min, 46 : 54 to 30 : 70 over 0.1 min, 30 : 70 to 1 : 99 over 5.9 min. MS data were processed using the instrument manufacturers software (MassLynx, version 4.1) and the xcms package (version 1.52.0)^24^ in the R statistical computing environment (version 3.4.1).^25^

Full methods are in the ESI.†

## Results and discussion

### Propranolol lipidation in liposomes composed of single lipids and binary mixtures

**Fig. 1:**
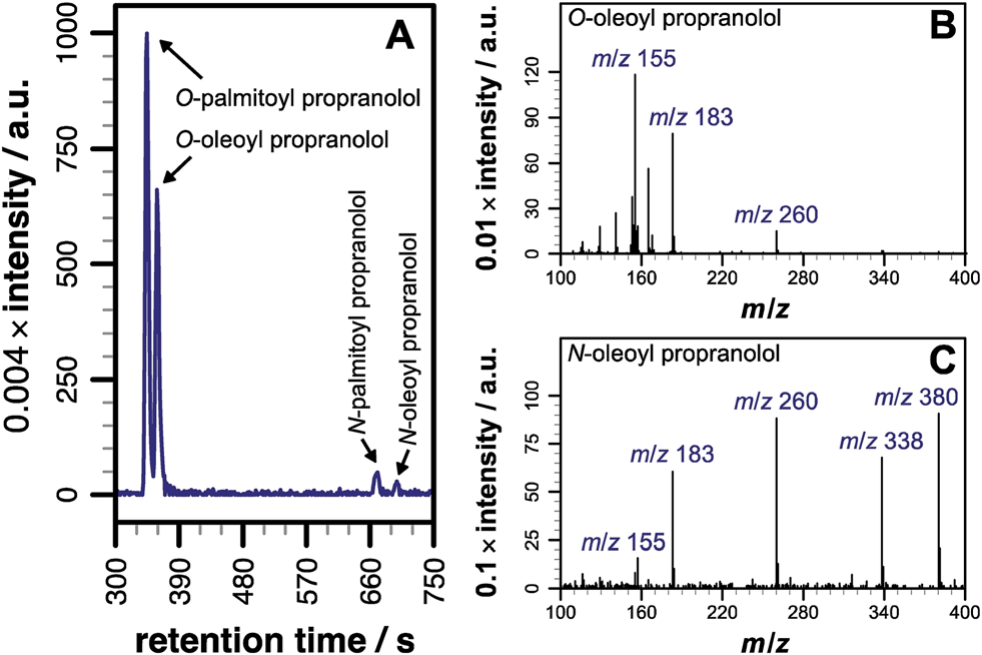
LC-MS analysis of lipidated products formed after addition of propranolol to POPC membranes. LC separation (A) was conducted on a C_18_ column using the protocol outlined in the methods section. (B) CID MSMS spectrum of *O*-oleoyl propranolol. (C) CID MSMS spectrum of *N*-oleoyl propranolol. Spectra for the corresponding palmitoyl products are in Fig. S2.† For assignments, see Tables S1–S3 and Fig. S3, S4.†

Our initial objective was to establish that incubation of propranolol (**1**) with liposomes could lead to the generation of lipidated propranolol products. We were able to obtain unambiguous evidence for propranolol lipidation through the use of liposomes with well-defined lipid compositions and comparison of the lipidated products with authentic standards prepared chemically. At 37 °C and pH 7.4, using liposomes composed of either 1-palmitoyl-2-oleoyl-*sn*-glycero-3-phosphocholine (POPC, Fig. 1a) or 1,2-dioleoyl-*sn*-glycero-3-phosphocholine (DOPC, Fig S1b†), lipidated propranolol (**2** and **3**, Scheme 1) was detectable in both cases, alongside increased levels of lysolipid. Lysolipids were found in both samples as a mixture of 1- and 2-oleoyl-*sn*-glycero-3-phosphocholine (OPC), in addition to 1- and 2-palmitoyl-*sn*-glycero-3-phosphocholine (PPC) in the case of POPC. The predominant products in both cases arose from a transesterification reaction involving the alcoholic group of propranolol to form the *O*-acyl product (**2**). *O*- and *N*-acyl modifications to propranolol were distinguishable by the shorter retention times for *O*-acyl propranolol derivatives in comparison to their *N*-acyl counterparts (Fig. 1a and S1†) and by their fragmentation patterns in tandem mass spectra produced by collision-induced decay (CID). The CID MSMS spectra of *O*-acyl propranolol (**2**) yielded a fragment of *m*/*z* 260, with species of *m*/*z* 155 and *m*/*z* 183 having the highest relative abundance (Fig. 1b and S1–S3, Table S1†). In contrast, *N*-acyl propranolol homologues (**3**) were particularly notable for the high relative abundance of fragments with an intact acyl chain, at *m*/*z* 338 and *m*/*z* 380 for *N*-oleoyl propranolol, alongside a high relative abundance for the fragment at *m*/*z* 260 corresponding to the loss of the acyl chain (Fig. 1c, S4, Tables S2 and S3†).

**Fig. 2:**
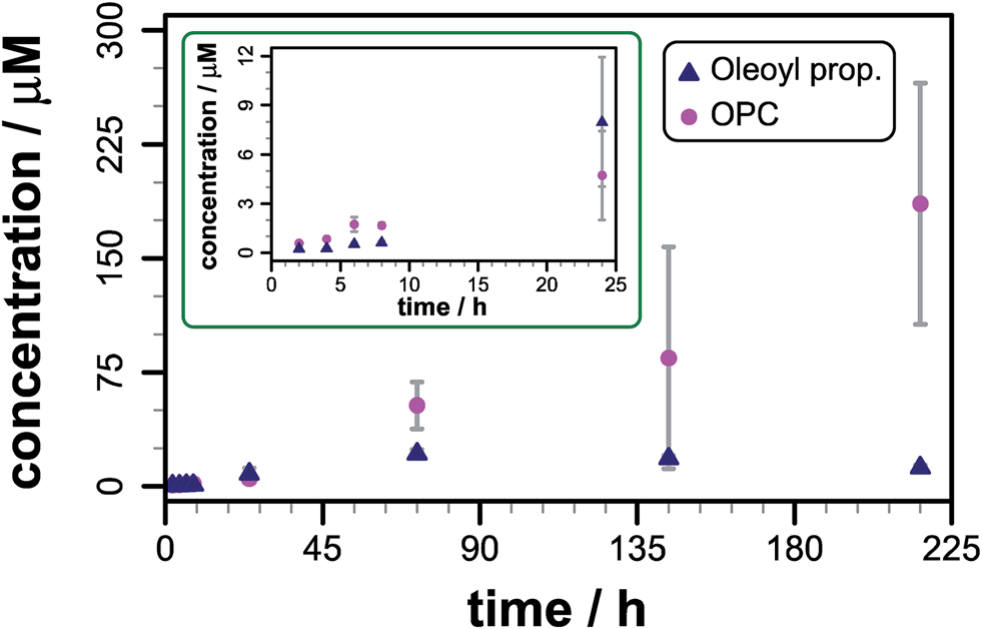
Product formation following the addition of propranolol (0.127 mM) to DOPC liposomes (1.27 mM) at pH 7.4, 37 °C. The total concentration of oleoyl propranolol (*N*- plus *O*-oleoyl) is shown as blue triangles. The lysolipid concentration, shown as magenta circles, is the concentration (1- plus 2-oleoyl) after subtraction of the lysolipid concentration in a control sample without propranolol. The inset shows a magnified view of the first data points. Errors are mean ± *σ*, *n* = 3.

After instrument calibration, using data from authentic standards to convert raw ion intensities to concentrations (Fig. S5, Table S4†), the concentrations of both total lipidated propranolol and lysolipid are seen to increase steadily over the first 48 h following mixing (Fig. 2). At these early time points, the predominant lipidated product is the *O*-acyl ester (Fig. S6†). The concentration of the *N*-acyl amide exhibits a small latency period before concentrations begin to increase, which indicates that the *N*-acyl product may be formed by *O* to *N* migration of the acyl group rather than direct aminolysis of the lipid. Such a migration has been described in the literature for propranolol ester derivatives in solution.^8^,^10^

The data are reproducible at time points up to 48 h, but show greater sample-to-sample variability over longer periods, particularly for the concentration of lysolipid. After 72 h the total content of lipidated propranolol decreases, the reasons for which are not currently understood, although hydrolysis or insolubility of one or both of the acyl derivatives are likely causes. Consequently, only data obtained within the first 48 h have been used for comparative analyses. As indicated in Fig. 1a, acyl transfer to propranolol from the lipid is expected to lead to the formation of a stoichiometric equivalent of lysolipid, which should lead to identical concentration changes for acyl propranolol and lysolipid, assuming that there are no competing processes. For DOPC, a linear fit to the data over the first 48 h gave a rate of acyl propranolol formation of 8.7 (±2.8) × 10^–5^ mM h^–1^, which was 0.6 (±0.3) times the rate of lysolipid formation in the same period. This indicates that either propranolol binding changes bilayer properties to facilitate hydrolysis, or that propranolol is able to catalyse lipid hydrolysis in addition to undergoing transesterification reactions. The lipidated propranolol products themselves may also promote lipid hydrolysis, for example by inducing changes to membrane properties that modify interfacial water activity, or by direct involvement in acid–base catalysis.

### Propranolol lipidation in lipid extracts

Having demonstrated lipidation of propranolol *in vitro*, it was of fundamental interest to probe whether this reaction also occurred *in cellulo*. In order to address this question propranolol was first incubated with liposomes composed of commercial lipid mixtures extracted from *E. coli* or liver cells (Fig. S7†) so that the likelihood of lipidation occurring in complex membranes could be assessed and the scale of the analytical challenge understood, given the likely complexity of the products.

**Fig. 3:**
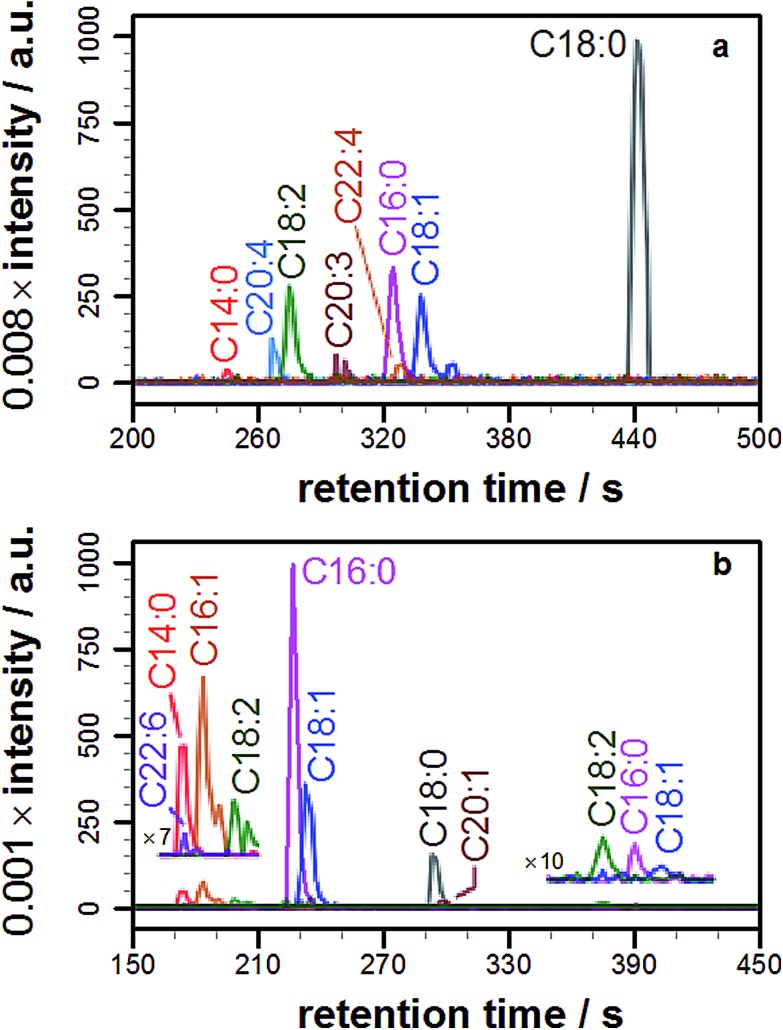
Overlaid extracted ion chromatograms (EICs) of the molecular ions corresponding to the lipidated propranolol species formed by incubation of propranolol with liver lipids. (a) Liposomes formed from bovine liver extract ([M + H]^+^; calculated *m*/*z* ± 6 ppm). (b) Acyl modified propranolol derivatives extracted from Hep G2 cells cultured in a medium containing 30 μM propranolol ([M + H]^+^; calculated *m*/*z* ± 7 ppm).

In both of these experiments, the ability to identify lipidation products was confirmed, with each extract yielding a series of acyl-modified derivatives (Fig. 3a, S8, Tables 1 and S5†) with relative abundances qualitatively in line with those expected based on the fatty acid profile of the extract, including modifications with cyclopropyl fatty acids for the *E. coli* extract and a high relative abundance for stearoyl-modified propranolol for the liver extract. Details of the lipid species identified are in the ESI (Tables S6 and S7†). Some products anticipated on the basis of the relative fatty acid abundances were not observed, including modification with C22:5. A number of reasons could account for the failure to observe these products, including their presence at concentrations below the detection limit, differences in the fatty acid profile of the commercial mixture from published data, peak overlap leading to ion suppression, and their presence in a form that is not reactive.

**Table 1:** Fatty acid profiles of bovine liver and Hep G2 phospholipids and the acylated propranolol derivatives observed by LCMS analysis after either propranolol (**1**) incubation with bovine liver extract liposomes, or the growth of Hep G2 cells for 72 h in the presence of propranolol (**1**)

Source	Fatty acid	Abund.^*a*^ (%)	Observed *m*/*z*^*b*^	Error^*c*^ (ppm)
Liver extract	14 : 0	—	470.3618	3.4
	16 : 0	11	498.3947	0.0
	18 : 0	29	526.4276	3.0
	18 : 1	10	524.4099	0.9
	18 : 2	8	522.3951	0.7
	20 : 3	4	548.4109	1.0
	20 : 4	16	546.3932	2.8
	22 : 4	2	574.4227	5.8
Hep G2 cells	14 : 0	6.4	470.3637	+0.6
	16 : 0	33.4	498.3940, 498.3937^*f*^	–1.4, –2.0^*f*^
	16 : 1	13.8	496.3762	–5.8
	18 : 0	5.7	526.4266	+1.2
	18 : 1	31.5^*d*^	524.4106, 524.4132^*f*^	+0.4, +5.4^*f*^
	18 : 2	0.7	522.3971, 522.3934^*f*^	+4.6, –2.4^*f*^
	20 : 1	0.7	552.4423	+1.1
	20 : 2^*e*^	—	550.4255	–0.9
	20 : 3^*e*^	—	548.4078	–4.7
	20 : 4^*e*^	1.0	546.3949	+0.4
	22 : 6	0.8	570.3966	+3.3

^*a*^Proportion (mol%) of each fatty acyl chain found in total bovine liver phospholipids^26^ or in lipids isolated from Hep G2 cells cultured in the absence of propranolol in a medium containing 10% foetal bovine serum.^27^ Some fatty acids reported in this study were not found as modifications to propranolol, including C18:3 (0.09 mol%), C20:5 (0.31 mol%) and C22:5 (0.92%).

^*b*^Observed *m*/*z* of acyl propranolol following fatty acyl transfer from the lipid. Data are for the *O*-acyl species unless otherwise stated.

^*c*^Error between observed and calculated *m*/*z* for [M + H]^+^ following transfer of this fatty acid to propranolol.

^*d*^Comprises 18:1*n*-7 (12.5%) and 18:1*n*-9 (19.0%).

^*e*^Not shown in Fig. 3b. Retention times: 192 s (20:4); 224 s (20:3); 247 s (20:2).

^*f*^ *N*-Acyl (amide).

### Propranolol lipidation *in cellulo*

With knowledge that acylated propranolol derivatives could be identified in complex mixtures of natural lipids, propranolol lipidation *in cellulo* was probed. The Hep G2 cell line was selected for this work in order to build on the data described above for liver cell extracts and because propranolol is known to induce phospholipidosis (PLD) in Hep G2 cells with an EC_50_ between 12.6 and 16 μM.^13^ In our hands, incubation of Hep G2 cells with 30 μM propranolol induced PLD as expected (Fig. S9†). By performing tests with POPC liposomes beforehand (Fig. S10†), the use of a chloroform/methanol mixture for the chemical extraction of Hep G2 cells was verified as the best approach for recovering both *O*- and *N*-acyl propranolol derivatives. When compared with the starting liposome mixture, a partial reduction in the relative intensity of *O*-oleoyl propranolol was found in the extracts, indicating a reduced extraction efficiency for propranolol modified with unsaturated fatty acyl groups. However, in the worst-case scenario all species could at least be partially recovered. Extraction of Hep G2 cells by this approach, followed by LC-MS analysis, identified a range of lipids (Fig. S11, Table S8†) alongside 14 different lipidated propranolol species (Fig. 3b, Table 1).

**Fig. 4:**
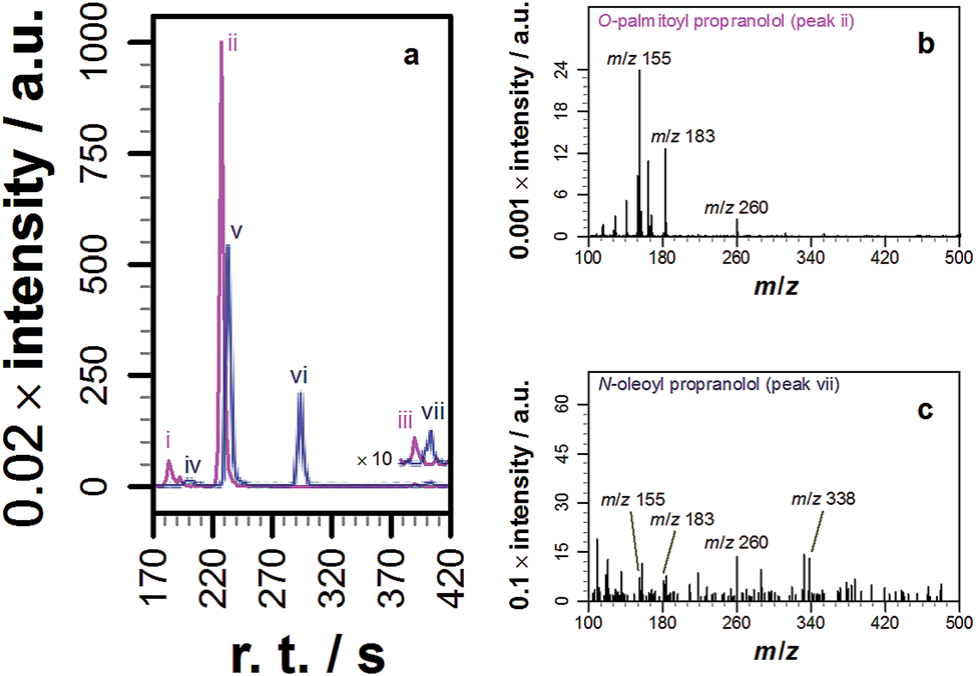
Tandem mass spectrometry analysis of samples extracted from Hep G2 cells cultured in a medium containing propranolol. (a) Extracted ion chromatograms (EICs) of product ions formed by targeted CID fragmentation of the ions with *m*/*z* 498.396 (magenta) and *m*/*z* 524.411 (blue), corresponding to [M + H]^+^ for palmitoyl propranolol and oleoyl propranolol respectively. A target mass window of ±4 *m*/*z* was used for CID. The chromatograms in (a) are the sum of the monoisotopic EICs for each ion in Fig. S3 and S4† with a mass window of ±8 ppm. Panels (b) and (c) show individual mass spectra corresponding to the indicated peaks in (a).

The predominant fatty acyl modifications to propranolol observed in the chromatograms, palmitoyl and oleoyl (Table 1), were those anticipated on the basis of published fatty acid profiles for Hep G2 cells cultured in the same medium, considered alongside the differences in extraction efficiency discussed above. The identities of some of the lipidated propranolol species were further confirmed by LC-MSMS analysis of the Hep G2 extract, with the palmitoyl- and oleoyl-modified propranolol parent species targeted for fragmentation (Fig. 4). As evidenced by the relatively early retention times and a high relative abundance of product ions with *m*/*z* 155 and *m*/*z* 183, the predominant products were *O*-acyl propranolol derivatives. Only trace quantities of the *N*-acyl species could be detected (Fig. 4, peaks iii and vii). The CID target mass window was sufficiently wide to confirm the presence of other related species in the mixture, including *O*-palmitoleoyl (Fig. 4, peak i), *O*-linoleoyl (peak iv) and *O*-stearoyl propranolol (peak vi).

In contrast to the experiments with extracted lipid mixtures, the reactivity of propranolol in Hep G2 membranes might be expected to produce a response that is influenced by the presence of lipid homeostasis in the cell. Indeed significant differences were found in the intensities of some lipids when comparing cells cultured with or without propranolol (Table S9†). These included decreases in the levels of some triglycerides and phosphatidylserine, and striking increases in the levels of ether-linked lipids, which would have the effect of reducing the ester content of the membrane that is transferrable in intrinsic lipidation reactions.

### The biological effects of drug lipidation

**Fig. 5:**
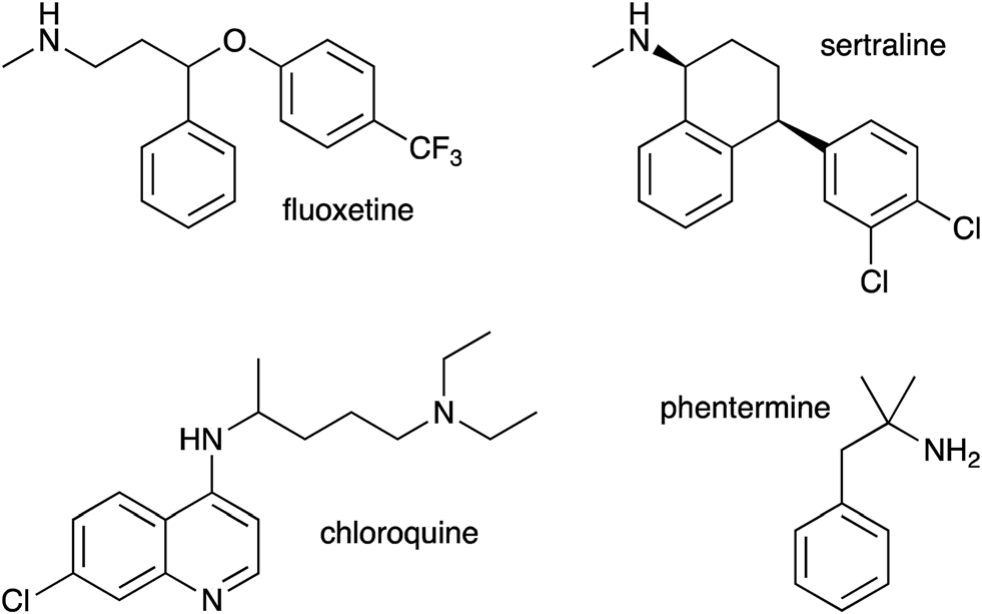
Drug molecules studied in this work in addition to propranolol (**1**).

**Fig. 6:**
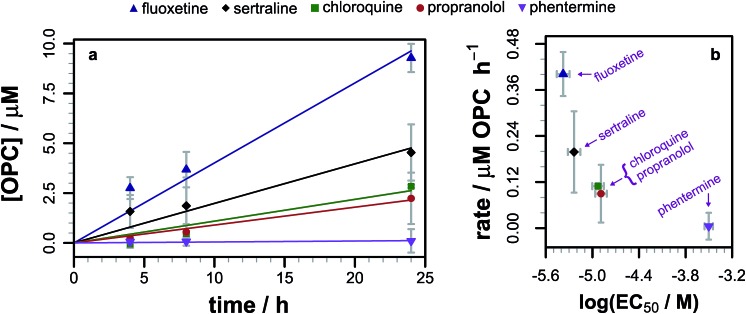
(a) Evolution of the 1/2-oleoyl-*sn*-glycero-3-phosphocholine (OPC) concentration in liposomes composed of DOPC (1.27 mM) following exposure to different drugs (0.127 mM) at pH 7.4 and 37 °C. Errors are mean ± *σ*, *n* = 3. Raw data are shown as points, linear fits to the reaction rate over the first 24 h as lines. (b) Relationship between the rate of lysolipid formation in DOPC and phospholipidosis activity^12^,^13^ for each of the drugs in part (a).

As we had evidence that propranolol reacts with membrane lipids both *in vitro* and *in vivo*, leading to the generation of lysolipids and a lipidated drug, it was of fundamental interest to establish whether this reactivity had the potential to induce detectable physiological effects. Previous work has established that some classes of drug linked with phospholipidosis are able to promote lipid hydrolysis in model systems *in vitro*, potentially by acting as phase transfer catalysts.^22^,^23^ We therefore explored whether propranolol and other compounds known to induce phospholipidosis (Fig. 5) were subject to lipidation in a model DOPC system or otherwise influence the levels of lysolipid, and examine the correlation between these activities and phospholipidosis activity. Interestingly, only propranolol underwent any lipidation reactions in DOPC membranes. However, all of the compounds with appreciable phospholipidosis activity yielded detectable increases in the initial rate of lipid hydrolysis over 24 h (Fig. 6a) compared to a control in the same conditions without compound. It may be the case that the amino group of propranolol is positioned in such a manner that it can promote the transesterification reactions of propranolol, but in the absence of proximal acyl group acceptor, the amino groups of the other drugs favour reactions involving interfacial water. Although there is a correlation between phospholipidosis activity and hydrolysis rates, with for example fluoxetine producing the fastest rate of lipid hydrolysis and having the lowest EC_50_ (Fig. 6b), the relationship is not simple, as indicated by the non-linearity in Fig. 6b.

It might be expected that both the lipidated drug and the lysolipid have the potential to disrupt lipid membranes as a consequence of their shape, which favours detergent-like activity. Both *N*-oleoyl and *N*-palmitoyl propranolol were found to have measurable micelle-forming behavior, with CMCs of 9 μM and 10 μM respectively (Fig. S12†). Addition of both of these *N*-acyl compounds at a concentration of 1 mol% to POPC liposomes loaded with the markers 8-aminonaphthalene-1,3,6-trisulfonic acid (ANTS, 12.5 mM) and *p*-xylene-bis-pyridinium bromide (DPX, 45 mM) resulted in the loss of membrane integrity with concomitant increase in fluorescence (Fig. S13†). Similar results were obtained for monooleoyl and monopalmitoyl PC. Parallel analyses using *O*-acyl species proved to be problematic due to the rapid hydrolysis of these species in the absence of membranes.

## Conclusions

This work has demonstrated the fundamental point that low molecular weight organic molecules are capable of generating lytic chemical processes in lipid membranes, including direct intermolecular reactions such as transesterification, or the promotion of other reactions such as hydrolysis. However, as evidenced by the reactivity of propranolol in comparison to drugs such as fluoxetine and sertraline, the selectivity for lipidation *vs.* hydrolysis is complex and most likely linked to a number of factors, including the molecular disposition in the membrane interface, the distribution profile (log *D*) and the p*K*_a_ values of any ionisable groups. Furthermore, it is clear that the activities observed *in vitro* are also likely to occur *in vivo*. This has been demonstrated directly by the observation of the products of lipidation reactions of propranolol in Hep G2 cells and indirectly by correlations between the hydrolysis promoting activity of drugs *in vitro* and their EC_50_ for phospholipidosis. In the case of propranolol it is also striking that the *O*-acyl esters, which normally rapidly rearrange to the *N*-acyl amide counterpart *in vitro*, exhibit increased stability in the membrane environment. This observation highlights the fact that there is much concerning the chemistry that occurs in the membrane interface that remains to be understood.

## Conflicts of interest

There are no conflicts to declare.

## Supplementary Material

Supplementary information
