# Supplementary material for: Lytic reactions of drugs with lipid membranes
Source: Chem Sci. 2018 Dec 3;10(3):674–80. doi: 10.1039/c8sc04831b (PMC6349070; doi:10.1039/c8sc04831b)
Supplement: Supplementary file 1 [file SC-010-C8SC04831B-s001.pdf]

Electronic Supplementary Material (ESI) for Chemical Science.
This journal is © The Royal Society of Chemistry 2018

Lytic Reactions of Drugs with Lipid Membranes: Supporting Information.

Hannah M. Britt[a], Clara A. G. Herrero[a], Paul W. Denny[b], Jackie A. Mosely[a] and John M. Sanderson[a]*

[a]Chemistry Department, Durham University, South Road, Durham, DH1 3LE, UK; [b]Department of Biosciences, Durham University, Stockton Road, Durham, DH1 3LE, UK.

Materials

Lipids were obtained as dry powders from Avanti Polar Lipids *via* Instruchemie B.V., The Netherlands. *E. coli* Extract Polar (catalogue number 100600P) is an extract of *E. coli* B (ATCC 11303) grown in Kornberg Minimal media at 37 °C and taken at ¾ log growth phase. Liver Polar Lipid Extract (Bovine, catalogue number 181108P) is derived from the total lipid extract by precipitation with acetone followed by extraction of the acetone insoluble material with diethyl ether. The LipidTOX™ phospholipidosis and steatosis detection kit (Molecular Probes brand) was purchased from Fisher Scientific, UK. Other compounds, including (±)-propranolol hydrochloride, fluoxetine hydrochloride, phentermine and chloroquine diphosphate salt were obtained from Sigma-Aldrich, Dorset, UK.

Methods

Liposome preparation: Liposomes were prepared by drying a solution of the lipid from a solution in chloroform to form a thin film around the side of a round bottomed flask. This film was then hydrated with buffer and after thorough vortex mixing was subjected to five freeze thaw cycles using liquid nitrogen and a warm water bath (40 °C). The vesicles were then extruded 10× through laser-etched polycarbonate membranes (Whatman, 100 nm pore size) at 50 °C using a thermobarrel extruder (Northern Lipids, Burnaby, Canada) under a positive pressure of N_2.

Liquid Chromatography-Mass Spectrometry (LC-MS): LC-MS and LC-MSMS data were acquired on a Synapt G2-S mass spectrometer (Waters Corp., UK) using electrospray ionisation with time-of-flight (ToF) mass analyser. Positive or negative ions were recorded in the range 50-2000 *m/z* with a scan time of 1.0 sec.

<u>Gradient 1</u>. For analysis of *E. coli* and liver lipid mixtures and samples extracted from Hep G2 cells, chromatography was conducted using a 3 µl sample injection on to an Acquity UPLC equipped with a CSH C_{18} 1.7 µm (2.1 x 150 mm) column (Waters Corp., UK) at 55 °C. The flow rate was 0.4 ml/min. The solvent gradient, using was H_2O:MeCN 4:6 (A) and MeCN:iPrOH 1:9 (B), with both solvents containing 10 mM NH_4HCO_2 + 0.1% formic acid, was as follows: 60:40 to 57:43 over 2 min, 57:43 to 50:50 over 0.1 min, 50:50 to 46:54 over 9.9 min, 46:54 to 30:70 over 0.1 min, 30:70 to 1:99 over 5.9 min, 1:99 to 60:40 over 0.1 min, 60:40 (isocratic) for 1.9 min. All solvent transitions used linear gradients. The electrospray parameters in positive mode were: capillary voltage, 2.5 kV; source temperature, 150 °C; sampling cone voltage, 50 V; source offset voltage, 30 V; desolvation temperature, 350 °C; cone gas flow, 60 l h^{-1}; desolvation gas flow, 600 l h^{-1}; nebuliser gas flow, 6 bar. The electrospray parameters in negative mode were the same, apart from the capillary voltage (2 kV) and the sampling cone voltage (30 V).

<u>Gradient 2</u>. For the analysis of mixtures of drugs with DOPC liposomes chromatography was conducted using a 3 µl sample injection on to an Acquity UPLC equipped with a BEH Phenyl 1.7 µm (2.1 x 50 mm) column. The flow rate was 0.4 ml/min. The solvent gradient (A:B), using H_2O (A) and MeCN (B), with both solvents containing 0.1% formic acid, was as follows: 95:5 over 0.5 min (isocratic), 95:5 to 5:95 over 7 min, 5:95 (isocratic) for 1.3 min, 5:95 to 95:5 over 0.1 min, 95:5 (isocratic) for 1.1 min. All solvent transitions used linear gradients. The electrospray parameters were: capillary voltage, 1 kV; source temperature, 150 °C; sampling cone voltage, 50 V; source offset voltage, 30 V; desolvation temperature, 350 °C; cone gas flow, 60 l h^{-1}; desolvation gas flow, 600 l h^{-1}; nebuliser gas flow, 6 bar.

CID MSMS was carried out in the trap region of a Synapt G2-S (Waters Ltd., UK). Desired precursor ions were isolated in the quadrapole, and subsequently underwent CID fragmentation ramping from 30 V to 50 V.

The data were processed using MassLynx software (version 4.1 SCN924) and the xcms LC-MS and GC-MS data analysis package (version 1.52.0)[1] in the R statistical computing environment (version 3.4.1)[2]. For complex lipid mixtures and Hep G2 extracts using xcms, the analysis consisted of two classes (with two samples per class): i. controls (without propranolol); and ii. samples extracted from Hep G2 cells grown in the presence of propranolol. Peak picking was done using the centWave method with the following parameters: ppm=8, peakwidth=c(0.4,12), snthresh=30, prefilter=c(5,2000), mzCenterFun="wMean", integrate=2, mzdiff=-0.005, fitgauss=TRUE, noise=700, firstBaselineCheck=TRUE. Peaks were grouped across samples with a bandwith (bw) of 2.5 and a minimum sample size (minsamp) of 2. Retention time correction was conducted using the symmetric algorithm. Peak areas were normalised between samples and runs using the total ion current across all scans. Data have only been reported for peaks where there is a clear change in intensity for both samples with propranolol *vs* both controls.

Calibration curves: Signal responses were determined by the least squares fitting of Gaussian functions to peak profiles from the extracted ion chromatograms (*m/z* for the molecular ion ± 8 ppm) of the ions of interest. Standard curves for signal response in relation to analyte concentration were generated by least squares fitting of a general logistic model (Equation S1) to data obtained using authentic standards of lysolipids, propranolol and acyl propranolol derivatives at known injection concentrations.

$$A_{\mathrm{calc}} = A_{\mathrm{u}}/1 + e^{-s(c-c_{0.5})} \qquad \text{Equation S1}$$

where A_{calc} is the calculated value, A_{u} is the maximum area, s is the steepness of the curve, c is the natural logarithm of the analyte concentration and $c_{0.5}$ is the natural logarithm of the concentration at half maximum. Fitting data are presented in Table S4 and Fig. S5.

Lipidation in liposomes: All liposome experiments were conducted at a lipid concentration of 1.27 mM. Drug molecules were used at a concentration of 0.127 mM. Samples at pH 7.4 were buffered using 10 mM sodium bicarbonate at contained NaCl at a concentration of 90 mM. Samples were made by adding compound solutions to pre-formed liposome dispersions before incubation in a sealed vial in either a temperature controlled thermal block or a thermostated shaking incubator. For analysis by LC-MS, a small volume (typically < 20 µl) of the reaction mixture was removed and diluted into MeCN/H_2O (1:1) in a sample vial to give a drug concentration of 1 µg/ml. The sample injection volume was 3 µl.

Phospholipidosis: LipidTOX™ assay. Hep G2 cells were grown until confluence in T-75 flasks (Nunc, ThermoFisher) in Dulbecco's modified Eagle medium (DMEM) supplemented with 10% (v/v) foetal bovine medium (Gibco/ThermoFisher) in an incubator at 37 °C under an atmosphere of 5% CO_2 at 95% humidity[3]. The growth medium was removed and the cells washed with phosphate buffered saline (PBS). 1 ml of Triplex solution was added and the flask gently agitated to detach the cells from the wall of the flask. Once the cells had separated, 9 ml of growing solution (minimum essential medium)[4] was added and a small sample (typically 10 µl) of the solution was used to determine cell concentration in a haemocytometer. If necessary, the cells were diluted to a concentration of 2×10^5 cells/ml with the growth medium.

Sterile glass coverslips were placed into each well of a 24-well plate. 1 ml of cell solution was added into the wells. The cells were left to attach overnight. After 24 h the medium was removed from the cells and 2×LipidTOX™ solution (200 µL) was added to each well, along with either 200 µL of a solution of the test compound in the growing medium at double the concentration to be tested, or 200 µL of medium. The LipidTOX™ dye as provided (1000×), was diluted 1:500 to obtain the 2×LipidTOX™ solution. The cells were incubated for 48 h under normal culture conditions before the growing medium was removed and the cells fixed using a solution of Hoeschst 33342 dye for nuclear labelling diluted 1:1000 with a 4% (v/v) solution of formaldehyde in PBS. The plate was left for half an hour at room temperature before the dye solution was removed and the wells washed with PBS buffer three times.

For microscopy, a small amount (< 5 µL) of the mounting medium VectaShield® was placed on a microscope slide. The coverslips with the cells were placed with tweezers face side down on the mounting solution. Any excess of VectaShield® was removed with a piece of paper. The coverslips were sealed with some nail

polish and a drop of oil placed on the lens before mounting the microscope slide. The cells were analysed by confocal microscopy using a Leica TCS SP5 confocal microscope in the Microscopy & Bioimaging Facility, Department of Biosciences, Durham University, UK. Hoeschst 33342 fluorescence was measured using a 352 nm excitation filter and a 461 nm emission filter. LipidTOX™ fluorescence was detected using a 595 nm excitation filter and a 615 emission filter. The scanner resolution was 8 bit, 1024×1024 pixels (98.4×98.4 µm), with a 95.5 µm pinhole. Fluorescence images were processed using LAS AF software (Leica Microsystems, version 3.0.2.16120).

Lipidation *in cellulo*: Hep G2 cells were grown until confluence using the method described above for the lipidation assay. Hep G2 cells (10^6) were added to each of four T-75 flasks and incubated at 37 °C, 5% CO_2, and 95% humidity overnight to adhere. The medium was removed and replaced with either fresh medium (5 ml) for two controls, or medium containing 30 µM propranolol (5 ml). After 72 h incubation, the medium was removed from flasks and replaced with PBS (5 ml). Cells were removed from surface by manual scraping, and washed three times with PBS. Solid cells were collected by 10 min centrifugation at 1000×g, and decanted into a glass tube for extraction. $CHCl_3$/MeOH (2:1; 3 ml) was added and the mixture shaken for 30 min before centrifugation to remove remaining solid. The $CHCl_3$/MeOH (2:1) solution was washed with H_2O (0.6 ml), isolated, and the solvent removed *in vacuo*. Samples were resuspended in ^{i}PrOH:MeCN:H_2O (2:1:1) for analysis by LCMS.

CMC determination: a stock solution of Rhodamine 6G was prepared in H_2O at 20 mg ml^{-1}. The stock rhodamine solution (1 ml) was added to a 1 ml cuvette, and the absorbance measured over the range 520 nm to 560 nm at 21 °C using a CARY100 UV-Visible spectrophotometer with Cary WinUV Scan Software 3.00. A 100 µm solution of the desired substrate in H_2O was added in 1 µl portions over the total concentration range 0.1 µM to 100 µM, and absorbance measurements repeated after each addition. Absorbance data were corrected for dilution[5,6].

Membrane stability assays: Stock solutions of 8-aminonaphthalene-1,3,6-trisulfonic acid (ANTS, 12.5 mM), *p*-xylene-bis-pyridinium bromide (DPX, 45 mM) and ammonium bicrabonate were prepared at pH 7.4 and their osmotic strengths measured using an Osmomat® 030 osmometer (Gonotec, Berlin, Germany). Their osmotic strengths were adjusted using either salt or water solutions at pH 7.4 to render them equal. Liposomes were prepared as described above, with phospholipid film rehydration conducted using a mixture of the 12.5 mm ANTS and 45.0mm DPX solutions. After extrusion, the external buffer was exchanged for the osmotically equivalent bicarbonate buffer using a PD-10 desalting column (GE Healthcare, UK). Liposomes (100 µl) were added to each well of a 96 well plate, along with the substrate (10 µl) of interest. The fluorescence emission at 530 nm was measured at one minute intervals following excitation at 360 nm using a Synergy H4 Plate Reader (BioTek, UK). Data were normalised to a positive control obtained in the presence of 20% Triton X-100.

Synthetic Procedures

General Methods. Reagents, including dry solvents were sourced from Fisher Scientific (Loughborough, UK) and Sigma-Aldrich (Dorset, UK), with the exception of dichloromethane (DCM), which was distilled from calcium hydride immediately before use. Purification was performed by flash column chromatography using a silica gel support (230–400 mesh, 40–60 µm) from Sigma–Aldrich (UK). Thin layer chromatography (TLC) was conduced using aluminium backed silica gel 60 F_{254} TLC plates (Merck Millipore, UK). TLC plates were visualised using either an ultraviolet (UV) lamp (254 nm), iodine chamber or by use of a dipping stain followed by heating. Dipping stains used included, phosphomolybdic acid (PMA) and $KMnO_4$. PMA stain consisted of PMA (6 g) and $Ce(SO_4)_2.4H_2O$ (3 g) dissolved in H_2SO_4 (15 ml) and H_2O (230 ml). $KMnO_4$ stain was prepared by dissolving $KMnO_4$ (1.5 g) and K_2CO_3 (10 g) in 10% NaOH solution (1 ml) and H_2O (200 ml). Distillation was performed using a Buchi Glass Oven B-585 Kugelrohr operating at a pressure between 0.2-2.0 Torr. NMR data were collected on a Bruker Avance-400 (at 400 MHz for ^{1}H; 100.6 MHz for ^{13}C) or Varian VNMRS (at 700 MHz for ^{1}H; 176 MHz for ^{13}C). NMR spectra were obtained in $CDCl_3$ (97% D, Goss Scientific Instruments µµtd., Cheshire, UK) and are reported in ppm using residual $CHCl_3$ at 7.26 ppm as the internal reference. ^{13}C NMR spectra are referenced to solvent as internal reference (77.23 ppm for $CDCl_3$). NMR data were processed using Mestrenova (Mestralab Research, version 10.0). Routine accurate mass characterisation was conducted on a LCT Premier XE (Waters Corp., UK) or QToF Premier (Waters Corp., UK). IR spectroscopy was performed on a Perkin Elmer Paragon 1000 FT-IR spectrometer with an ATR attachment operating through the wavenumber range 400 cm^{-1} and 4000 cm^{-1}. IR data were processed using the instrument software.

Synthesis of *N*-tert-Butoxycarbonyl Propranolol. To propranolol (0.048 g, 0.18 mmol) and NEt_3 (0.127 ml, 0.92 mmol) in DCM (2 ml) was added Boc_2O (0.080 mg, 0.37 mmol) and the solution stirred for 12 hours. The solution was diluted with DCM (8 ml), washed with saturated aqueous NH_4Cl (3 × 10 ml), dried over $MgSO_4$ and filtered. The solvent was removed *in vacuo* to give the crude product as a yellow oil which was purified by Kugelrohr distillation to give the desired product (0.051 g, 79%): ^{1}H NMR (400 MHz; $CDCl_3$) δ = 1.23 (d, J = 5.8 Hz, 6H, $H_{20,21}$), 1.57 (s, 9H, $H_{16–18}$), 3.50-3.55 (m, 2H, H_{13}), 4.01-4.09 (m, 1H, H_{19}), 4.17-4.21 (m, 2H, $H_{11,12}$), 4.24-29 (m, 1H, H_{11}), 5.13 (s, 1H, OH), 6.86 (d, J = 8.0 Hz, 1H, H_9), 7.33-7.38 (m, 1H, H_8), 7.43-7.45 (m, 1H, H_7), 7.47-7.51 (m, 2H, $H_{1,2}$), 7.80 (d, J = 8.0 Hz, 1H, H_3), 8.22 (d, J = 8.0 Hz, 1H, H_6); ^{13}C NMR (100 MHz; $CDCl_3$) δ = 20.5 ($C_{20,21}$), 28.5 ($C_{16–18}$, 47.1 (C_{13}), 48.8 (C_{19}), 69.9 (C_{11}), 72.1 (C_{12}), 80.9 (C_{15}), 104.9 (C_9) 120.6 (C_7), 121.6 (C_6), 125.1 (C_5), 125.4 (C_1), 125.9 (C_8), 126.4 (C_2), 127.6 (C_3), 134.5 (C_4), 154.3 (C_{10}), 158.8 (C_{14}); IR (neat) v_{max}/cm^{-1} 1692, 2953, 2983, 3476; LRMS (ESI) m/z 360.2 [M+H]$^+$; HRMS (ESI) calculated for $C_{21}H_{30}NO_4$ [M+H]$^+$ 360.2175, found 360.2168. Data are consistent with the literature[7].

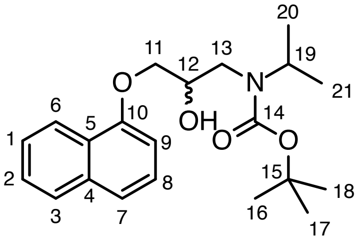

Synthesis of *O*-Palmitoyl *N*-tert-Butoxycarbonyl Propranolol. To *N*-butoxycarbonyl propranolol (0.031g, 0.09 mmol) in dry DCM (2 ml) was added pyridine (0.008ml, 0.10mmol) and palmitoyl chloride (0.029 ml, 0.10 mmol). The solution was stirred for 12 hours at room temperature, diluted with DCM (8 ml) and washed with saturated aqueous NH_4Cl solution (3 × 10 ml). The organic layer was dried over MgSO4, filtered and the solvent removed *in vacuo*. The crude product was purified by flash column chromatography chromatography with eluent EtOAc/MeOH/14% aq. NH_3 (98:1:1) to yield the desired product as a yellow oil (0.035 g, 65%): ^{1}H NMR (400 MHz; $CDCl_3$) δ = 0.88 (t, J = 8.0 Hz, 3H, H_{28}), 1.11-1.33 (m, 30H, H_{Alkyl}), 1.51 (s, 9H, H_{32-34}), 1.61 (quint, J = 24.0 Hz, 2H, H_{15}), 2.32 (q, J = 12.0 Hz, 2H, H_{14}), 3.39-3.52 (m, 2H, H_{29}), 3.58-3.66 (m, 1H, H_{35}), 4.19-4.22 (m, 2H, $H_{11,12}$), 4.28-4.33 (m, 1H, H_{11}), 6.78 (d, J = 8.0 Hz, 1H, H_9), 7.34 (t, J = 8.0 Hz, 1H, H_8), 7.41-7.44 (m, 1H, H_7), 7.45-7.51 (m, 2H, $H_{1,2}$), 7.79 (d, J = 8.0 Hz, 1H, H_3), 8.21 (d, J = 8.0 Hz, 1H, H_6); ^{13}C NMR (100 MHz; $CDCl_3$) δ = 14.1 (C_{28}), 22.7 (C_{Alkyl}), 24.9 (C_{Alkyl}), 28.4 (C_{Alkyl}), 29.1 (C_{Alkyl}), 29.2 (C_{Alkyl}), 29.3 (C_{Alkyl}), 29.6 (C_{Alkyl}), 29.7 (C_{Alkyl}), 31.9 (C_{Alkyl}), 34.3 (C_{Alkyl}), 47.1 (C_{29}), 48.8 (C_{35}), 68.1 (C_{11}), 72.8 (C_{12}), 80.0 (C_{31}),

104.6 (C_9) 120.6 (C_7), 121.9 (C_6), 125.4 (C_5), 125.5 (C_1), 125.7(C_8), 126.4 (C_2), 127.4 (C_3), 134.5 (C_4), 154.3 (C_{10}), 173.4 (C_{30}), 178.8 (C_{13}); IR (neat) v_{max}/cm^{-1} 1600, 1715, 2916, 2998; LRMS (ESI) *m/z* 598.4 $[M+H]^+$; HRMS (ESI) calculated for $C_{37}H_{60}NO_5$ $[M+H]^+$ 598.4471, found 598.4571.

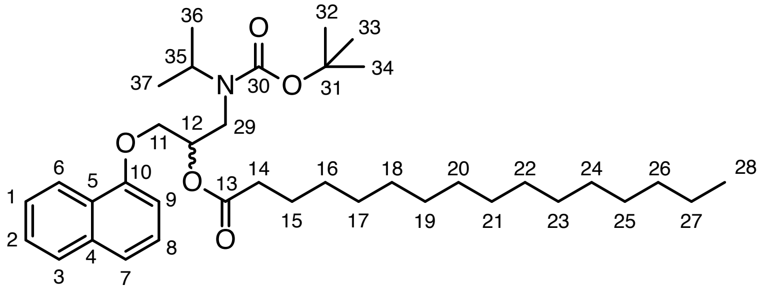

Synthesis of *O*-Oleoyl *N*-tert-Butoxycarbonyl Propranolol. This was synthesised by the same method as *O*-palmitoyl *N*-butoxycarbonyl propranolol, replacing palmitoyl chloride with the same number of equivalents of oleoyl chloride to give the product (0.035 g, 63%) as a yellow oil. ^1H NMR (400 MHz; $CDCl_3$) δ = 0.88 (t, J = 8.0 Hz, 3H, H_{30}), 1.18-1.36 (m, 24H, H_{Alkyl}), 1.51 (s, 9H, $H_{34–36}$), 1.97-2.01 (m, 4H, $H_{20,23}$), 2.15 (q, J = 12.0 Hz, 2H, H_{15}), 2.88 (t, 2H, J = 8.0 Hz, H_{14}), 3.39-3.52 (m, 2H, H_{31}), 3.58-3.66 (m, 1H, H_{37}), 4.19-4.22 (m, 2H, $H_{11,12}$), 4.28-4.33 (m, 1H, H_{11}), 5.30-5.35 (m, 2H, $H_{21,22}$), 6.78 (d, J = 8.0 Hz, 1H, H_9), 7.36 (t, J = 8.0 Hz, 1H, H_8), 7.42-7.44 (m, 1H, H_7), 7.45-7.51 (m, 2H, $H_{1,2}$), 7.80 (d, J = 8.0 Hz, 1H, H_3), 8.22 (d, J = 8.0 Hz, 1H, H_6); ^{13}C NMR (100 MHz; $CDCl_3$) δ = 14.1 (C_{30}), 22.7 (C_{Alkyl}), 24.9 (C_{Alkyl}), 28.4 (C_{Alkyl}), 29.1 (C_{Alkyl}), 29.2 (C_{Alkyl}), 29.3 (C_{Alkyl}), 29.6 (C_{Alkyl}), 29.7 (C_{Alkyl}), 31.9 (C_{Alkyl}), 34.3 (C_{Alkyl}), 47.1 (C_{31}), 48.8 (C_{37}), 68.1 (C_{11}), 72.8 (C_{12}), 80.0 (C_{33}), 104.6 (C_9) 120.6 (C_7), 121.9 (C_6), 125.4 (C_5), 125.5 (C_1), 125.7(C_8), 126.4 (C_2), 127.4 (C_3), 130.5 (C_{21}), 130.7 (C_{22}), 134.5 (C_4), 154.3 (C_{10}), 173.9 (C_{32}), 178.8 (C_{13}); IR (neat) v_{max}/cm^{-1} 1598, 1741, 2915, 2929; LRMS (ESI) *m/z* 624.5 $[M+H]^+$; HRMS (ESI) calculated for $C_{39}H_{62}NO_5$ $[M+H]^+$ 624.4628, found 624.4710.

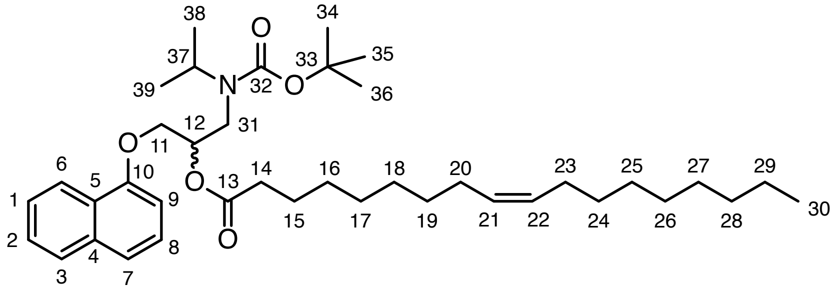

Synthesis of *O*-Palmitoyl Propranolol Hydrochloride. Aqueous HCl (37%, 0.6 ml) was added dropwise to *O*-palmitoyl *N*-butoxycarbonyl propranolol (0.06g, 0.09 mmol) in DCM (0.6 ml) and the solution stirred for 1.5 hours. Volatile materials were removed *in vacuo* to yield the HCl salt of the desired compound (0.044g, 98%): ^1H NMR (400 MHz; $CDCl_3$) δ = 0.88 (t, J = 8.0 Hz, 3H, H_{28}), 1.11-1.33 (m, 30H, H_{Alkyl}), 1.61 (quint, J = 24.0 Hz, 2H, H_{15}), 2.32 (q, J = 12.0 Hz, 2H, H_{14}), 3.39-3.52 (m, 2H, H_{29}), 3.58-3.66 (m, 1H, H_{30}), 4.19-4.22 (m, 2H, $H_{11,12}$), 4.28-4.33 (m, 1H, H_{11}), 6.78 (d, J = 8.0 Hz, 1H, H_9), 7.34 (t, J = 8.0 Hz, 1H, H_8), 7.41-7.44 (m, 1H, H_7), 7.45-7.51 (m, 2H, $H_{1,2}$), 7.79 (d, J = 8.0 Hz, 1H, H_3), 8.21 (d, J = 8.0 Hz, 1H, H_6), 9.70 (s, 1H, NH); ^{13}C NMR (100 MHz; $CDCl_3$) δ = 14.1 (C_{28}), 22.7 (C_{Alkyl}), 24.9 (C_{Alkyl}), 28.4 (C_{Alkyl}), 29.1 (C_{Alkyl}), 29.2 (C_{Alkyl}), 29.3 (C_{Alkyl}), 29.6 (C_{Alkyl}), 29.7 (C_{Alkyl}), 31.9 (C_{Alkyl}), 34.3 (C_{Alkyl}), 47.1 (C_{29}), 48.8 (C_{30}), 68.1 (C_{11}), 72.8 (C_{12}), 104.6 (C_9) 120.6 (C_7), 121.9 (C_6), 125.4 (C_5), 125.5 (C_1), 125.7(C_8), 126.4 (C_2), 127.4 (C_3), 134.5 (C_4), 154.3 (C_{10}), 173.4 (C_{13}); IR (neat) v_{max} $/cm^{-1}$ 1745, 2856, 2924; LRMS (ESI) *m/z* 498.4 $[M+H]^+$; HRMS (ESI) calculated for $C_{32}H_{52}NO_3$ $[M+H]^+$ 498.3947, found 498.3943.

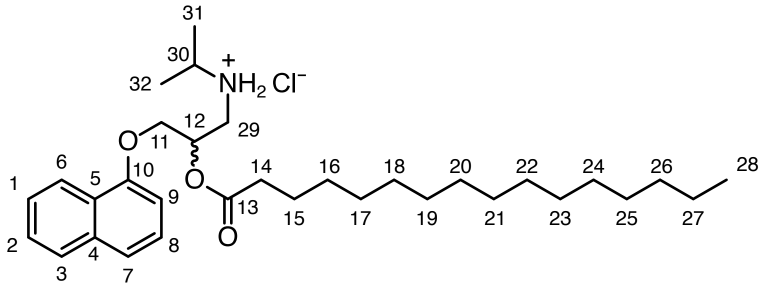

Synthesis of *O*-Oleoyl Propranolol Hydrochloride. This was prepared in an identical manner to *O*-palmitoyl propranolol using *O*-oleoyl *N*-butoxycarbonyl propranolol (0.06g, 0.09 mmol) as the starting material to give the hydrochloride salt of the desired compound (0.045 g, 96%): ^{1}H NMR (400 MHz; $CDCl_3$) δ = 0.88 (t, *J* = 8.0 Hz, 3H, H_{30}), 1.18-1.36 (m, 24H, H_{Alkyl}), 1.97-2.01 (m, 4H, $H_{20,23}$), 2.15 (q, *J* = 12.0 Hz, 2H, H_{15}), 2.88 (t, 2H, *J* = 8.0 Hz, H_{14}), 3.39-3.52 (m, 2H, H_{31}), 3.58-3.66 (m, 1H, H_{32}), 4.19-4.22 (m, 2H, $H_{11,12}$), 4.28-4.33 (m, 1H, H_{11}), 5.30-5.35 (m, 2H, $H_{21,22}$), 6.78 (d, *J* = 8.0 Hz, 1H, H_9), 7.36 (t, *J* = 8.0 Hz, 1H, H_8), 7.42-7.44 (m, 1H, H_7), 7.45-7.51 (m, 2H, $H_{1,2}$), 7.80 (d, *J* = 8.0 Hz, 1H, H_3), 8.22 (d, *J* = 8.0 Hz, 1H, H_6), 9.58 (s, 1H, NH); ^{13}C NMR (100 MHz; $CDCl_3$) δ = 14.1 (C_{30}), 22.7 (C_{Alkyl}), 24.9 (C_{Alkyl}), 28.4 (C_{Alkyl}), 29.1 (C_{Alkyl}), 29.2 (C_{Alkyl}), 29.3 (C_{Alkyl}), 29.6 (C_{Alkyl}), 29.7 (C_{Alkyl}), 31.9 (C_{Alkyl}), 34.3 (C_{Alkyl}), 47.1 (C_{31}), 48.8 (C_{32}), 68.1 (C_{11}), 72.8 (C_{12}), 104.6 (C_9) 120.6 (C_7), 121.9 (C_6), 125.4 (C_5), 125.5 (C_1), 125.7(C_8), 126.4 (C_2), 127.4 (C_3), 130.5 (C_{21}), 130.7 (C_{22}), 134.5 (C_4), 154.3 (C_{10}), 175.2 (C_{13}); IR (neat) ν_{max}/cm^{-1} 1744, 2859, 2930; LRMS (ESI) *m/z* 524.4 $[M+H]^+$; HRMS (ESI) calculated for $C_{34}H_{54}NO_3$ $[M+H]^+$ 524.4104, found 524.4103.

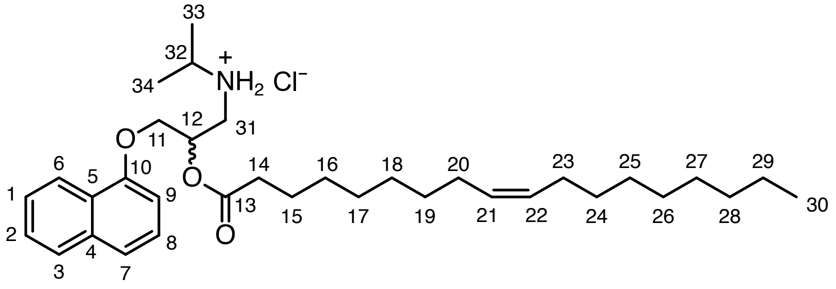

Synthesis of *O,N*-Dipalmitoyl Propranolol. To propranolol (0.050g, 0.17mmol) in dry DCM (4 ml) was added pyridine (0.015 ml, 0.19 mmol) and palmitoyl chloride (0.112 ml, 0.37 mmol). The solution was stirred for 12 hours at room temperature, diluted with DCM (6 ml) and washed with saturated aqueous NH_4Cl solution (3 × 10 ml). The organic layer was dried over $MgSO_4$, filtered, and solvent removed *in vacuo*. Purification by flash column chromatography using eluent EtOAc/MeOH/14% aq. NH_3 (98:1:1) yielded the desired compound as a yellow oil (0.105g, 84%): ^{1}H NMR (400 MHz; $CDCl_3$) δ = 0.87 (t, *J* = 7.0 Hz, 6H, $H_{28,45}$), 1.11-1.36 (m, 54H, H_{Alkyl}), 1.57-1.65 (m, 4H, $H_{15,32}$), 2.29-2.41 (m, 6H, $H_{14,31}$), 3.42 (dd, *J* = 6.9, 14.3 Hz, 0.75 H, H_{29}), 3.62 (dd, *J* = 4.0, 15.8 Hz, 0.25 H, H_{29}), 3.82 (dd, *J* = 5.3, 14.3 Hz, 1H, H_{29}), 4.08–4.15 and 4.42–4.47 (m, 0.75H and 0.25H, H_{46}), 4.22–4.32 (m, 2H, H_{11}), 5.42–5.47 and 5.52–5.58 (m, 0.25H and 0.75H, H_{12}), 6.79-6.82 (m, 1H, H_9), 7.33-7.38 (m, 1H, H_8), 7.43 (d, *J* = 8.0 Hz, 1H, H_7), 7.44–7.50 (m, 2H, $H_{1,2}$), 7.75–7.82 (m, 1H, H_3), 8.18–8.21 (m, 1H, H_6); ^{13}C NMR (100 MHz; $CDCl_3$) δ = 14.1 ($C_{28,45}$), 20.3 (C_{Alkyl}), 20.7 (C_{Alkyl}), 21.1 (C_{Alkyl}), 21.9 (C_{Alkyl}), 22.7 (C_{Alkyl}), 24.7 (C_{Alkyl}), 25.0 (C_{Alkyl}), 25.5 (C_{Alkyl}), 29.0 (C_{Alkyl}), 29.3 (C_{Alkyl}), 29.6 (C_{Alkyl}), 31.9 (C_{Alkyl}), 33.9 ($C_{14,31}$), 34.4 ($C_{14,31}$), 41.3 (C_{15}), 48.5 (C_{46}), 68.5 (C_{11}), 70.7 (C_{11}), 71.6 (C_{12}), 72.5 (C_{12}), 104.8 (C_9), 120.5 (C_7), 121.4 (C_6), 121.9 (C_6), 125.1 (C_5), 125.2 (C_5), 125.6 (C_1), 125.5 (C_1), 125.6 (C_8), 125.8 (C_2), 125.9 (C_8), 126.3 (C_4), 127.4 (C_2), 127.6 (C_4), 134.4 (C_4), 154.3 (C_{10}), 173.4 (C_{30}), 173.9 (C_{13}); IR (neat) ν_{max}/cm^{-1} 1676, 1745, 2855, 2926; LRMS (ESI) *m/z* 736.6 $[M+H]^+$; HRMS (ESI) calculated for $C_{48}H_{82}NO_4$ $[M+H]^+$ 736.6244, found 736.6256.

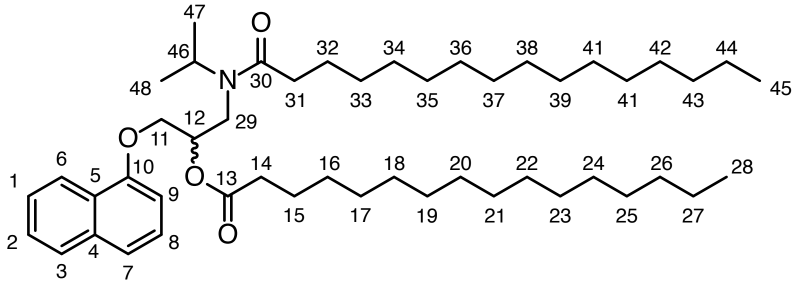

Synthesis of *N*-Palmitoyl Propranolol. *O,N*-dipalmitoyl propranolol (0.050 g, 0.14 mmol) and K_2CO_3 (0.059 g, 0.43 mmol) were stirred in MeOH (1.5 ml) for 2 hours prior to solvent removal *in vacuo*. The resulting solid was resuspended in MeOH (6 ml) and $^{c}H_2SO_4$ (0.12 ml) and refluxed for 1 hour to methylate excess fatty acid. The solution was diluted with DCM (10 ml), washed with saturated $NaHCO_3$ solution until neutral, dried over $MgSO_4$, filtered, and the solvent removed *in vacuo*. Purification of the crude product by flash column chromatography using eluent EtOAc/MeOH/14% aq. NH_3 (98:1:1) yielded the desired compound as a white solid (0.049g, 70%): ^{1}H NMR (400 MHz; CDCl3) δ = 0.87 (t, *J* = 7.0 Hz, 3H, H_{29}), 1.11-

1.36 (m, 54H, H_{Alkyl}), 1.59-1.65 (m, 2H, H_{16}), 2.32-2.38 (m, 2H, H_{15}), 3.51 (dd, *J* = 1.5, 14.6 Hz, 1H, H_{13}), 3.81 (dd, *J* = 8.4, 14.6 Hz, 1H, H_{13}), 4.04-4.29 (m, 4H, $H_{11,12,30}$), 5.74 (s, 1H, OH), 6.82-6.88 (m, 1H, H_9), 7.38-7.43 (m, 1H, H_8), 7.46–7.59 (m, 3H, $H_{1,2,7}$), 7.81–7.87 (m, 1H, H_3), 8.21–8.28 (m, 1H, H_6); ^{13}C NMR (100 MHz; $CDCl_3$) δ = 14.4 (C_{29}), 20.3 (C_{Alkyl}), 20.7 (C_{Alkyl}), 21.3 (C_{Alkyl}), 21.7 (C_{Alkyl}), 21.8 (C_{Alkyl}), 22.7 (C_{Alkyl}), 24.7 (C_{Alkyl}), 25.0 (C_{Alkyl}), 25.3 (C_{Alkyl}), 25.6 (C_{Alkyl}), 29.1 (C_{Alkyl}), 29.2 (C_{Alkyl}), 30.3 (C_{15}), 46.1 (C_{13}), 50.3 (C_{30}), 69.8 (C_{11}), 72.5 (C_{12}), 104.8 (C_9), 120.7 (C_7), 121.5 (C_6), 125.2 (C_5), 125.4 (C_1), 126.0 (C_8), 126.3 (C_2), 127.7 (C_3), 134.5 (C_4), 154.07 (C_{10}), 176.9 (C_{14}); IR (neat) ν_{max}/cm^{-1} 1745, 2914, 2926, 3391; LRMS (ESI) *m/z* 498.4 $[M+H]^+$; HRMS (ESI) calculated for $C_{32}H_{52}NO_3$ $[M+H]^+$ 498.3947, found 498.3943.

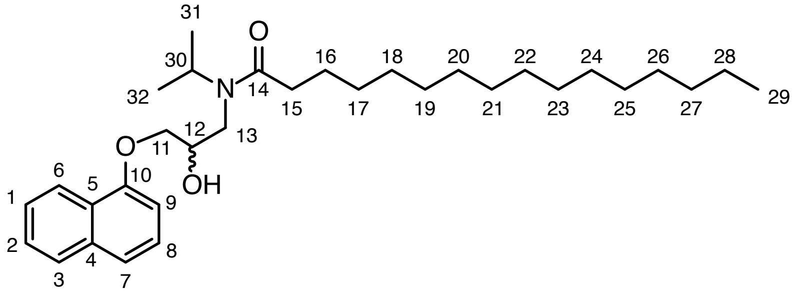

Synthesis of *O,N*-Dioleoyl Propranolol. This was prepared using the same method as *O,N*-dipalmitoyl propranolol using oleoyl chloride (0.112 ml, 0.37 mmol) in place of palmitoyl chloride, to give the desired compound as a yellow oil (0.110 g, 82%): ^{1}H NMR (400 MHz; $CDCl_3$) δ = 0.88 (t, 6H, *J* = 8.0 Hz, $H_{30,49}$), 1.13-1.37 (m, 46H, H_{Alkyl}), 1.55-1.67 (m, 4H, $H_{15,34}$), 1.97-2.03 (m, 8H, $H_{20,23,39,42}$), 2.31-2.51 (m, 4H, $H_{14,33}$), 3.38 and 3.66 (dd, *J* = 6.9/14.3 Hz and 4.0/15.8 Hz, 0.75H and 0.25H, H_{31}), 3.85 (dd, J = 5.3, 14.3 Hz, 1H, H_{31}), 4.08–4.17 and 4.48–4.52 (m, 0.75H and 0.25H, H_{50}), 4.24–4.34 (m, 2H, H_{11}), 5.27-5.37 (m, 4H, $H_{21,22,40,41}$), 5.42–5.47 and 5.55–5.58 (m, 0.25H and 0.75H, H_{12}), 6.79-6.88 (m, 1H, H_9), 7.33-7.38 (m, 1H, H_8), 7.40–7.53 (m, 3H, $H_{1,2,7}$), 7.77–7.83 (m, 1H, H_3), 8.19–8.22 (m, 1H, H_6); ^{13}C NMR (100 MHz; $CDCl_3$) δ = 14.1 ($C_{30,49}$), 21.1 (C_{Alkyl}), 22.7 (C_{Alkyl}), 25.5 (C_{Alkyl}), 27.1 (C_{Alkyl}), 27.2 (C_{Alkyl}), 29.9 (C_{Alkyl}), 29.1 (C_{Alkyl}), 29.2 (C_{Alkyl}), 29.4 (C_{Alkyl}), 29.7 (C_{Alkyl}), 29.8 (C_{Alkyl}), 41.3 (C_{31}), 48.4 (C_{50}), 49. (C_{50}), 53.4 ($C_{14,33}$), 68.5 (C_{11}), 70.7 (C_{12}), 71.7 (C_{12}), 72.5 ($C_{21,22,40,41}$), 104.7 (C_9), 104.8 (C_9), 120.5 (C_7), 121.2 (C_7), 121.5 (C_6), 121.9 (C_6), 125.1 (C_5), 125.5 (C_1), 125.8 (C_8), 126.3 (C_2), 126.6 (C_8), 127.4 (C_4), 127.6 (C_2), 129.8 (C_4), 129.9 (C_4), 134.4 (C_4), 154.3 (C_{10}), 173.3 (C_{32}), 173.9 (C_{13}); IR (neat) ν_{max}/cm^{-1} 1652, 1746, 2915, 2986, 2930; LRMS (ESI) *m/z* 788.7 $[M+H]^+$; HRMS (ESI) calculated for $C_{52}H_{86}NO_4$ $[M+H]^+$ 788.6557, found 788.6573.

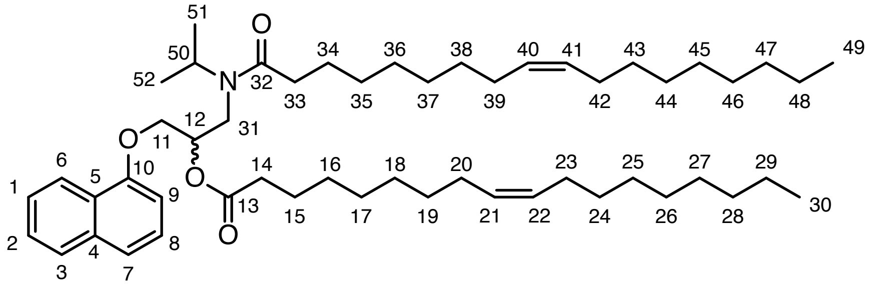

Synthesis of *N*-Oleoyl Propranolol. This was prepared from *O,N*-dioleoyl propranolol using the same procedure as that used for the preparation of *N*-palmitoyl propranolol from *O,N*-dipalmitoyl propranolol to give the desired compound as a yellow oil (0.051g, 70 %): 1H NMR (400 MHz; $CDCl_3$) δ = 0.88 (t, 3H, *J* = 8.0 Hz, H_{31}), 1.09 (d, *J* = 6.6 Hz, 6H, $H_{33,34}$), 1.19-1.39 (m, 20H, H_{Alkyl}), 1.59-1.72 (m, 2H, H_{16}), 194-2.08 (m, 4H, $H_{21,24}$), 2.86 and 3.05 (quin, *J* = 20.0 Hz, 0.75H and 1.25H, H_{15}), 3.54 (dd, *J* = 1.5, 14.6 Hz, 1H, H_{13}), 3.75 (dd, *J* = 8.4, 14.6 Hz, 1H, H_{13}), 4.11-4.35 (m, 4H, $H_{11,12,32}$), 5.27-5.57 (m, 2H, $H_{22,23}$); 5.74 (s, 1H, OH), 6.80-6.87 (m, 1H, H_9), 7.37-7.40 (m, 1H, H_8), 7.40–7.51 (m, 3H, $H_{1,2,7}$), 7.79–7.83 (m, 1H, H_3), 8.20 (d, *J* = 6.0 Hz, 1H, H_6); ^{13}C NMR (100 MHz; $CDCl_3$) δ = 14.1 (C_{31}), 20.7 (C_{Alkyl}), 21.5 (C_{Alkyl}), 22.7 (C_{Alkyl}), 23.0 (C_{Alkyl}), 25.0 (C_{Alkyl}), 25.4 (C_{Alkyl}), 27.2 (C_{Alkyl}), 29.0 (C_{Alkyl}), 29.1 (C_{Alkyl}), 29.2 (C_{Alkyl}), 29.3 (C_{Alkyl}), 29.5 (C_{Alkyl}), 29.7 (C_{Alkyl}), 31.9(C_{Alkyl}), 33.8 (C_{Alkyl}), 34.0 (C_{Alkyl}), 46.2 (C_{13}), 47.3 (C_{13}), 48.6 (C_{32}), 49.1 (C_{32}), 67.8 (C_{11}), 69.9 (C_{11}), 71.7 (C_{12}), 72.5 (C_{12}), 104.8 (C_9), 120.6 (C_7), 121.6 (C_7), 121.9 (C_6), 125.2 (C_6), 125.4 (C_5), 125.7 (C_1), 125.9 (C_8), 126.3 (C_2), 127.4 (C_8), 127.6 (C_2), 129.7 (C_3), 130.0 ($C_{22,23}$), 134.5 (C_4), 154.0 (C_{10}), 173.6 (C_{14}), 176.4 (C_{14}); IR (neat) ν_{max}/cm^{-1} 1628, 2858, 2930, 3415; LRMS (ESI) *m/z* 524.4 $[M+H]^+$; HRMS (ESI) calculated for $C_{34}H_{54}NO_3$ $[M+H]^+$ 524.4104, found 524.4103.

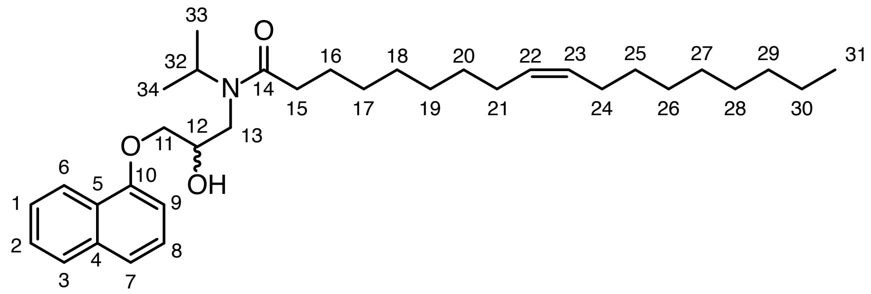
O
N
OH
O
1
2
3
4
5
6
7
8
9
10
11
12
13
14
15
16
17
18
19
20
21
22
23
24
25
26
27
28
29
30
31
32
33
34

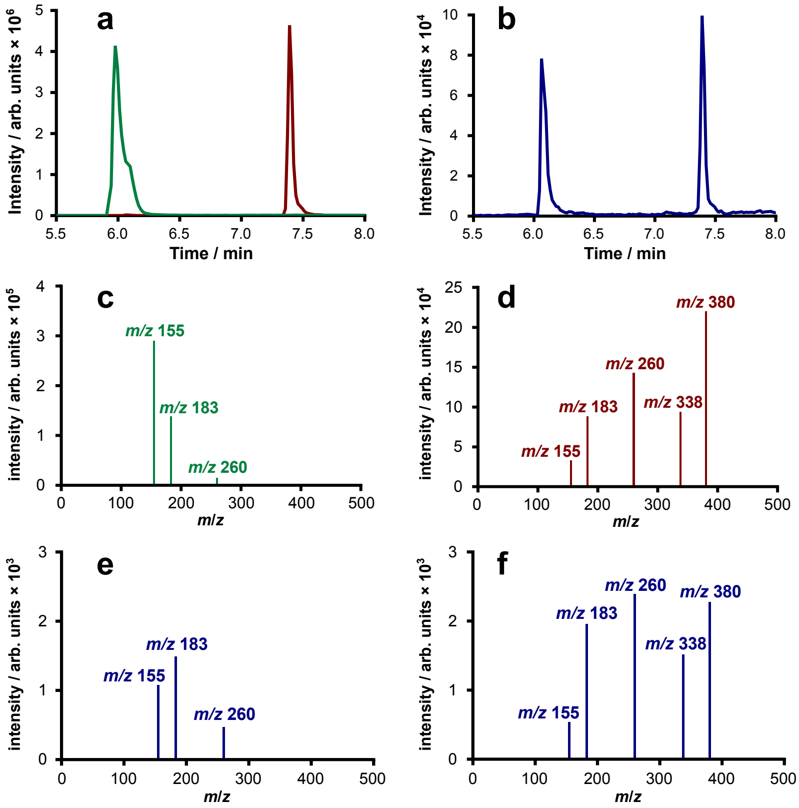

Fig. S1. **a**, **b**: extracted ion chromatograms (EICs) corresponding to *m/z* 524.410 (± 5 ppm) for oleoyl propranolol ($[M+H]^+$) in synthetic *O*-oleoyl propranolol (**a**, green), synthetic *N*-oleoyl propranolol (**a**, red), and a mixture of propranolol with DOPC liposomes (**b**). **c** to **f**: CID MSMS spectra of oleoyl propranolol species corresponding to **a** and **b**: **c**, synthetic *O*-oleoyl propranolol; **d**, synthetic *N*-oleoyl propranolol; **e**, the peak of retention time 6.1 min in the DOPC/propranolol mixture; and **f**, the peak of retention time 7.4 min in the DOPC/propranolol mixture.

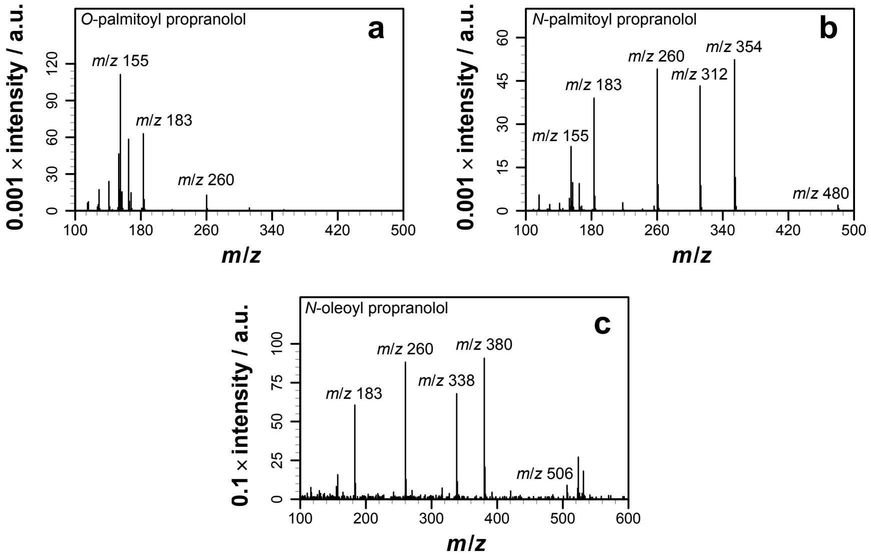

Fig. S2. LC-MS analysis of lipidated products formed after addition of propranolol to POPC membranes. LC separation was conducted on a BEH C_{18} column using the protocol outlined in the methods section. For assignments, see Tables S1-S3 and Fig. S3-S4. **a**, CID MSMS of *O*-palmitoyl propranolol. **b**, CID MSMS of *N*-palmitoyl propranolol. **c**, CID MSMS of *N*-oleoyl propranolol (full scan version of the spectrum in Fig. 1d of the main paper).

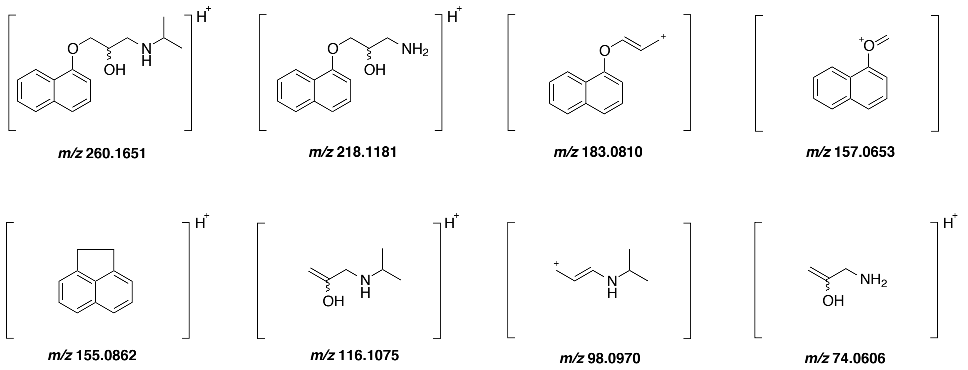

Fig. S3. CID MSMS product ion assignments, involving loss of the acyl chain, for *N*-oleoyl propranolol, *N*-palmitoyl propranolol, *O*-oleoyl propranolol and *O*-palmitoyl propranolol.

Table S1. MSMS fragments observed from CID MSMS of protonated *O*-palmitoyl propranolol *m/z* 498.3961 and protonated *O*-oleoyl propranolol *m/z* 524.4111.

**Theoretical *m/z***	**Molecular Formula**	**Accuracy *O*-palmitoyl propranolol (ppm)**	**Accuracy *O*-oleoyl propranolol (ppm)**	**RDB**
74.0606	$[C_3H_8NO]^+$	-5.4	1.4	0.5
98.0970	$[C_6H_{12}N]^+$	1.0	0.0	1.5
116.1075	$[C_6H_{14}NO]^+$	-1.7	0.0	0.5
155.0861	$[C_{12}H_{11}]^+$	-2.6	-1.9	7.5
157.0653	$[C_{11}H_9O]^+$	-1.9	-2.5	7.5
183.0810	$[C_{13}H_{11}O]^+$	-1.6	-2.2	8.5
218.1181	$[C_{13}H_{16}NO_2]^+$	-3.7	-6.4	6.5
260.1651	$[C_{16}H_{22}NO_2]^+$	-2.3	-1.9	6.5

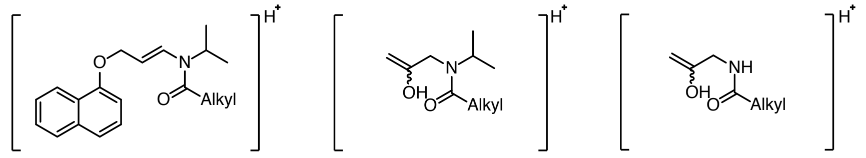

alkyl:

**palmitoyl**	***m/z* 480.3835**	***m/z* 354.3364**	***m/z* 312.2896**
**oleoyl**	***m/z* 506.3998**	***m/z* 380.3529**	***m/z* 338.3059**

Fig. S4. CID MSMS product ion assignments for *N*-oleoyl propranolol and *N*-palmitoyl propranolol involving retention of the acyl chain.

Table S2. MSMS fragments observed following CID of protonated *N*-oleoyl propranolol.

Obs. *m/z*	Molecular Formula	Accuracy (ppm)	RDB
74.0603	$[C_3H_8NO]^+$	-10.8	0.5
98.0959	$[C_6H_{12}N]^+$	-11.2	1.5
116.1074	$[C_6H_{14}NO]^+$	-0.9	0.5
155.0859	$[C_{12}H_{11}]^+$	-0.6	7.5
157.0654	$[C_{11}H_9O]^+$	+0.6	7.5
183.0808	$[C_{13}H_4O]^+$	-1.1	8.5
218.1173	$[C_{13}H_{16}NO_2]^+$	-4.1	6.5
260.1645	$[C_{16}H_{22}NO_2]^+$	-2.7	6.5
338.3053	$[C_{21}H_{40}NO_2]^+$	-1.8	2.5
380.3523	$[C_{24}H_{46}NO_2]^+$	-1.3	2.5
506.3989	$[C_{34}H_{52}NO_2]^+$	-1.0	9.5

Table S3. MSMS fragments observed following CID of protonated *N*-palmitoyl propranolol.

Observed *m/z*	Molecular Formula	Accuracy (ppm)	RDB
74.0609	$[C_3H_8NO]^+$	+2.7	0.5
98.0970	$[C_6H_{12}N]^+$	-2.0	1.5
116.1073	$[C_6H_{14}NO]^+$	-2.6	0.5
155.0860	$[C_{12}H_{11}]^+$	-0.6	7.5
157.0653	$[C_{11}H_9O]^+$	-0.6	7.5
183.0808	$[C_{13}H_4O]^+$	-1.1	8.5
218.1178	$[C_{13}H_{16}NO_2]^+$	-2.3	6.5
260.1648	$[C_{16}H_{22}NO_2]^+$	-1.2	6.5
312.2896	$[C_{19}H_{38}NO_2]^+$	-2.2	1.5
354.3364	$[C_{22}H_{44}NO_2]^+$	-2.5	1.5
480.3835	$[C_{32}H_{50}NO_2]^+$	-0.6	8.5

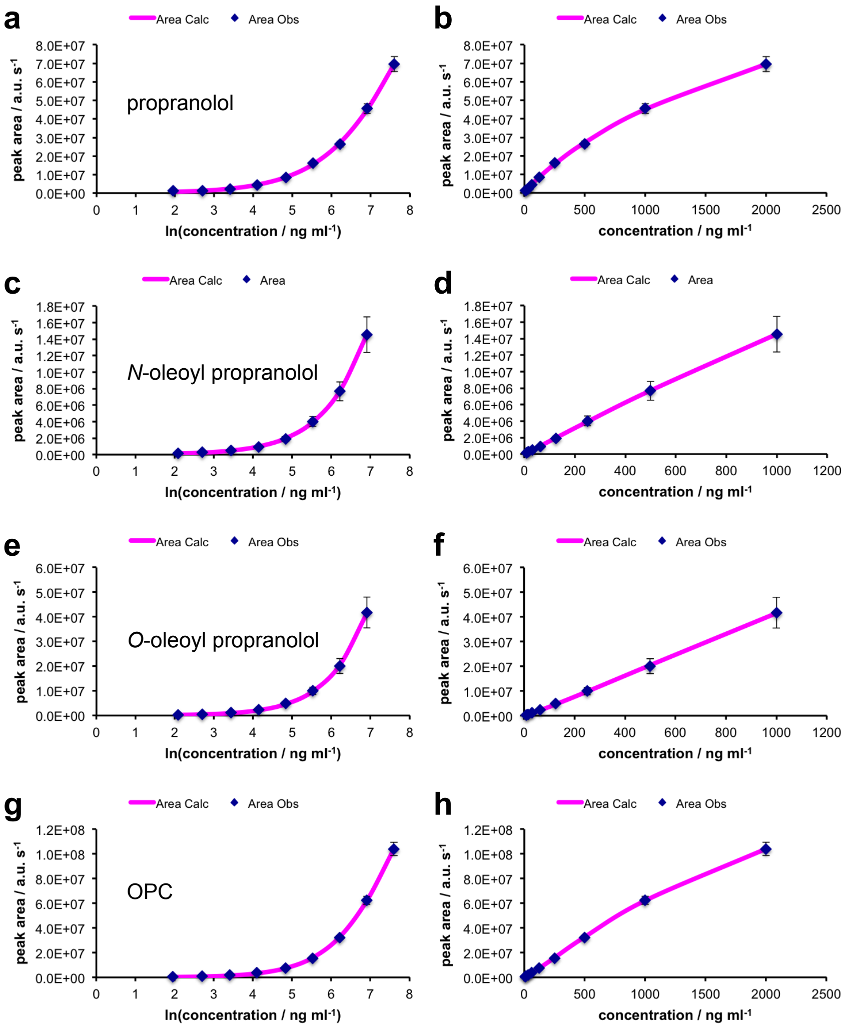

Fig. S5. Calibration curves showing the variation of chromatographic peak area response over a range of sample vial concentrations of authentic samples (with fixed injection volume): **a/b**, propranolol; **c/d**, *N*-oleoyl propranolol; **e/f**, *O*-oleoyl propranolol; **g/h**, oleoyl PC (summed areas for both 1- and 2-oleoyl). Raw data are shown as points, the outputs from fitting a general logistic model (equation S1) to the data as lines. Each pair (**a/b**, **c/d** *etc*) shows the same data but differ in how the *x*-axis is plotted. Error bars are mean ± σ, *n*=3.

Table S4. Fitting parameters from intensity *vs* concentration calibrations (Equation S1).

**System**	A_u **/ a.u. s**$^{-1}$	$c_{0.5}$	s	**% error** [a]
OPC	229792594	7.76	1.16	5.3
N-Oleoyl Propranolol	98967519	8.62	1.03	14.9
O-Oleoyl Propranolol	364592082	8.75	1.11	4.8
Propranolol	181325593	8.12	0.91	9.3

[a] Based on 2 standard deviations.

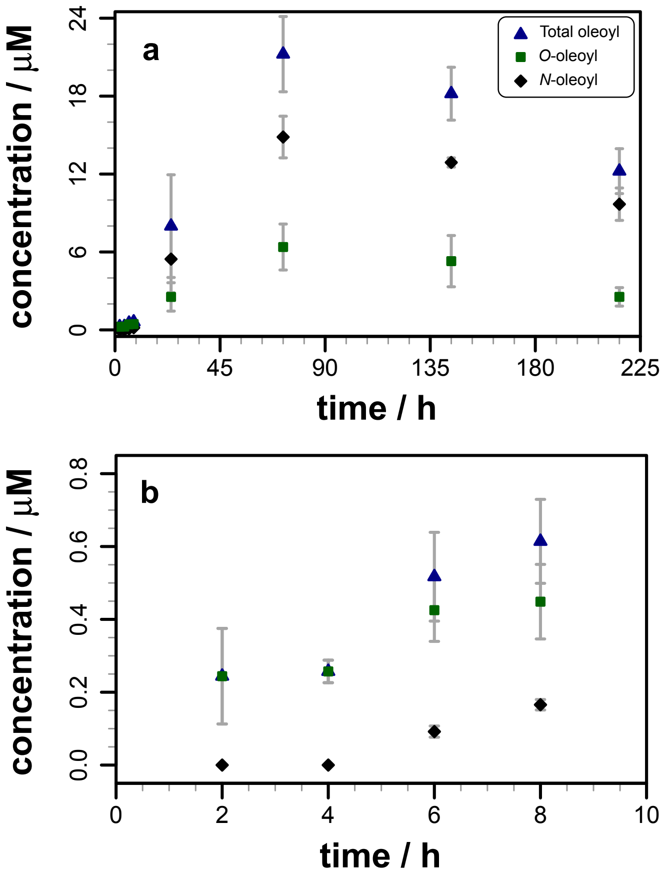

Fig. S6. Product formation under physiological conditions (37 °C, pH 7.4) following the addition of propranolol to DOPC liposomes: *N*-oleoyl propranolol (black diamonds), *O*-oleoyl propranolol (green squares) and total oleoyl propranolol (blue triangles). **a**, all data. **b**, an expanded view of the data at early time points. Error bars are mean ± σ, *n*=3.

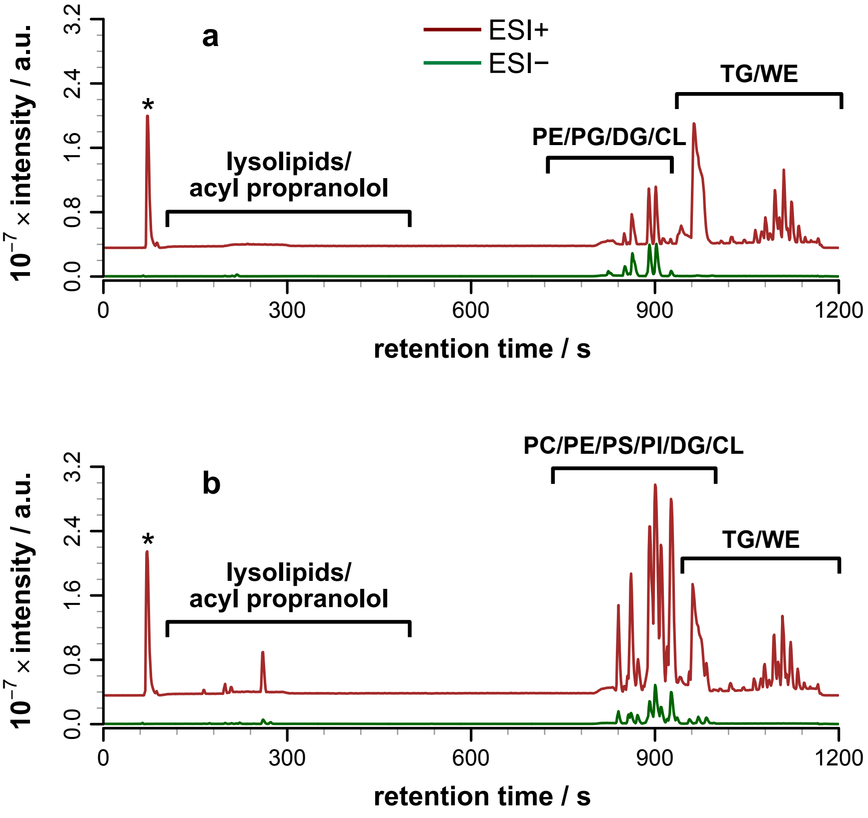

Fig. S7. Incubations of propranolol with complex lipid mixtures. Panels **a** and **b** show the base peak normalised chromatograms of commercial lipid extracts from *E. coli* (**a**) and liver cells (**b**) following incubation with propranolol. Positive mode ESI is shown in red and negative mode ESI in green. Positive mode chromatograms are offset by 0.35 arbitrary units (a.u.) on the *y*-axis. The propranolol retention time (corresponding to the injection peak) is indicated by an asterisk. LC separation was conducted on a CSH C_{18} column using Gradient 1 outlined in the methods section. Key: CL, cardiolipin; Cer, ceramide; DG, diacylglycerol; PE, phosphatidylethanolamine; PG, phosphatidylglycerol; PI, phosphatidylinositol; PS, phosphatidylserine; SM, sphingomyelin; TG, triacylglycerol; WE, wax ester.

Table S5. Fatty acid profiles of *E. coli* (ATCC 11303) lipid extracts and acylated propranolol derivatives observed by LCMS analysis following propranolol incubation at pH 7.4 and 37 °C with liposomes prepared from *E. coli* lipid extract.

Fatty Acid	*E. coli* (ATCC 11303) (%) [a]	Calc. *m/z*	Obs. *m/z*	$[M+H]^+$ Error (ppm)	R.T. / min
14:0	1.6	470.3634	470.3617	3.7	4.1
16:0	37.4	498.3947	498.3956	1.8	5.4
16:1	5.1	496.3791	496.3805	2.9	4.3
cyclo 17:0	18.9	510.3947	510.3960	2.5	5.1
18:0	0.5	526.4260	-	-	-
18:1	31.0	524.4104	524.4106	0.4	5.1
cyclo 19:0	5.3	538.4260	538.4264	0.7	6.9

[a] proportion (%) of each fatty acyl chain found in lipids extracted from *E. coli* (ATCC 11303) in the absence of propranolol[8].

Table S6. Phospholipid and lysolipid species identified within the commercial *E. coli* lipid extract.

**Observed *m/z***	**Theoretical *m/z***	**$[M+H]^+$ error (ppm)**	**Assignment [a]**
452.2743	452.2782	3.6	LPE(16:0)
464.2751	464.2782	3.6	LPE(17:1)
478.2907	478.2939	3.6	LPE(18:1)
660.4620	660.4610	1.5	PE(30:1)
686.4742	686.4766	3.5	PE(32:2)
690.5097	690.5079	2.6	PE(32:0)
693.4714	693.4712	0.3	PG(30:0)
716.5262	716.5236	3.6	PE(34:1)
720.4960	720.4973	1.8	PE(36:5)‡
721.5034	721.5025	1.2	PG(32:0)
730.5397	730.5392	0.7	PE(35:1)
735.5152	735.5181	3.9	PG(33:0)
746.5138	746.5130	1.1	PE(38:6)‡
748.5286	748.5286	0.0	PE(38:6)† / PE(38:5)‡
749.5318	749.5338	2.7	PG(34:0)
762.4703	762.4715	1.6	PE(37:7(OH))
772.4933	772.4923	1.3	PE(39:8)
777.4683	777.4712	3.7	PG(37:7)
798.6023	798.6018	0.6	PE(40:2)
804.5884	804.5912	3.5	PE(42:6)† / PE(42:5)‡
805.6296	805.6328	4.0	PG(39:0)†
811.5496	811.5494	0.2	PG(39:4)
817.5013	817.5025	1.5	PG(40:8)
852.6826	852.6851	2.9	PE(45:3)† / PE(45:2)‡
873.6237	873.6226	1.3	PG(42:2(OH))
922.7305	922.727	3.8	PE(49:3)
1101.9003	1101.9043	3.6	PG(58:0(OH))

[a] † indicates the presence of alkyl ether substituent; ‡ indicates the presence of alkenyl ether (plasmalogen) substituent.

Table S7. Phospholipid and lysolipid species identified within commercial Bovine liver lipid extract.

Observed *m/z*	Theoretical *m/z*	$[M+H]^+$ Error (ppm)	Assignment [a]
438.2988	438.2979	2.1	LPE(16:0)‡
452.2762	452.2772	2.2	LPE(16:1)
466.3274	466.3292	3.9	LPE(18:0)‡
478.2938	478.2928	2.1	LPE(18:2)
482.3228	482.3241	2.7	LPE(18:0)
502.2926	502.2928	0.4	LPE(20:4)
508.3756	508.3762	1.2	LPC(18:0)‡
518.3239	518.3241	0.4	LPC(18:3)
544.3383	544.3398	2.8	LPC(20:4)
546.3540	546.3554	2.6	LPC(20:3)
568.3392	568.3398	1.1	LPC(22:6)
597.3057	597.3035	3.7	LPI(18:2)
601.3324	601.3348	4.0	LPI(18:0)
690.5065	690.5069	0.6	PE(32:1)
694.5036	694.5018	2.6	PC(28:0(OH))
698.5127	698.5119	1.1	PE(34:4)† / PE(34:3)‡
718.5381	718.5382	0.1	PE(34:1)
748.5272	748.5276	0.5	PE(38:6)‡
748.6210	748.6215	0.7	PC(34:0)†
750.5461	750.5432	3.9	PE(38:6)† / PE(38:5)‡
756.5531	756.5538	0.9	PC(34:3)
756.5930	756.5902	3.7	PE(38:2)‡
768.5548	768.5538	1.3	PE(38:4)
770.6049	770.6058	1.2	PC(36:3)†/PC(36:2)‡
772.5859	772.5851	1.0	PE(38:2)
784.4915	784.4912	0.4	PE(40:10)
790.5955	790.5957	0.3	PE(38:1(OH))
808.5826	808.5851	3.1	PC(38:5)
828.5161	828.5174	1.6	PE(42:10(OH))
828.6099	828.6113	1.7	PC(38:3(OH))
830.5680	830.5695	1.8	PC(40:8)
832.6788	832.6790	0.2	PE(42:0)
837.5488	837.5488	0.0	PI(34:1)
842.6614	842.6634	2.4	PC(40:2)
845.5183	845.5175	0.9	PI(35:4)
846.6953	846.6947	0.7	PC(40:0)
850.5942	850.5957	1.8	PC(40:6(OH))
853.6163	853.6165	0.2	PI(36:0)†
856.7183	856.7154	3.4	PC(42:2)†
862.6303	862.6321	2.1	PC(42:6)
863.5669	863.5644	2.9	PI(36:2)
869.5526	869.5539	1.5	PI(38:6)† / PI(38:5)‡
884.7485	884.7467	2.0	PC(44:2)†
885.5472	885.5488	1.8	PI(38:5)
887.5615	887.5644	3.3	PI(38:4)
889.5770	889.5801	3.5	PI(38:3)
891.5959	891.5957	0.2	PI(38:2)
905.6475	905.6478	0.3	PI(40:2)† / PI(40:1)‡
915.5927	915.5957	3.3	PI(40:4)
931.5306	931.5331	2.7	PI(42:10)

[a] † indicates the presence of alkyl ether substituent; ‡ indicates the presence of alkenyl ether (plasmalogen) substituent.

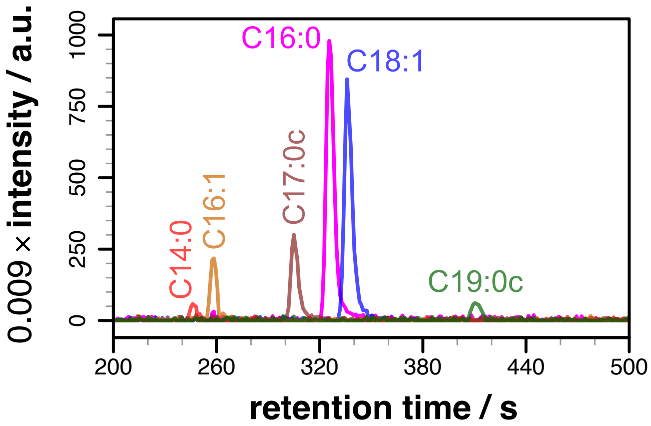

Fig. S8. Extracted ion chromatograms (EICs) of the molecular ions ($[M+H]^+$) corresponding to the lipidated propranolol species formed by incubation of propranolol with liposomes formed from *E. coli* polar lipid extract.

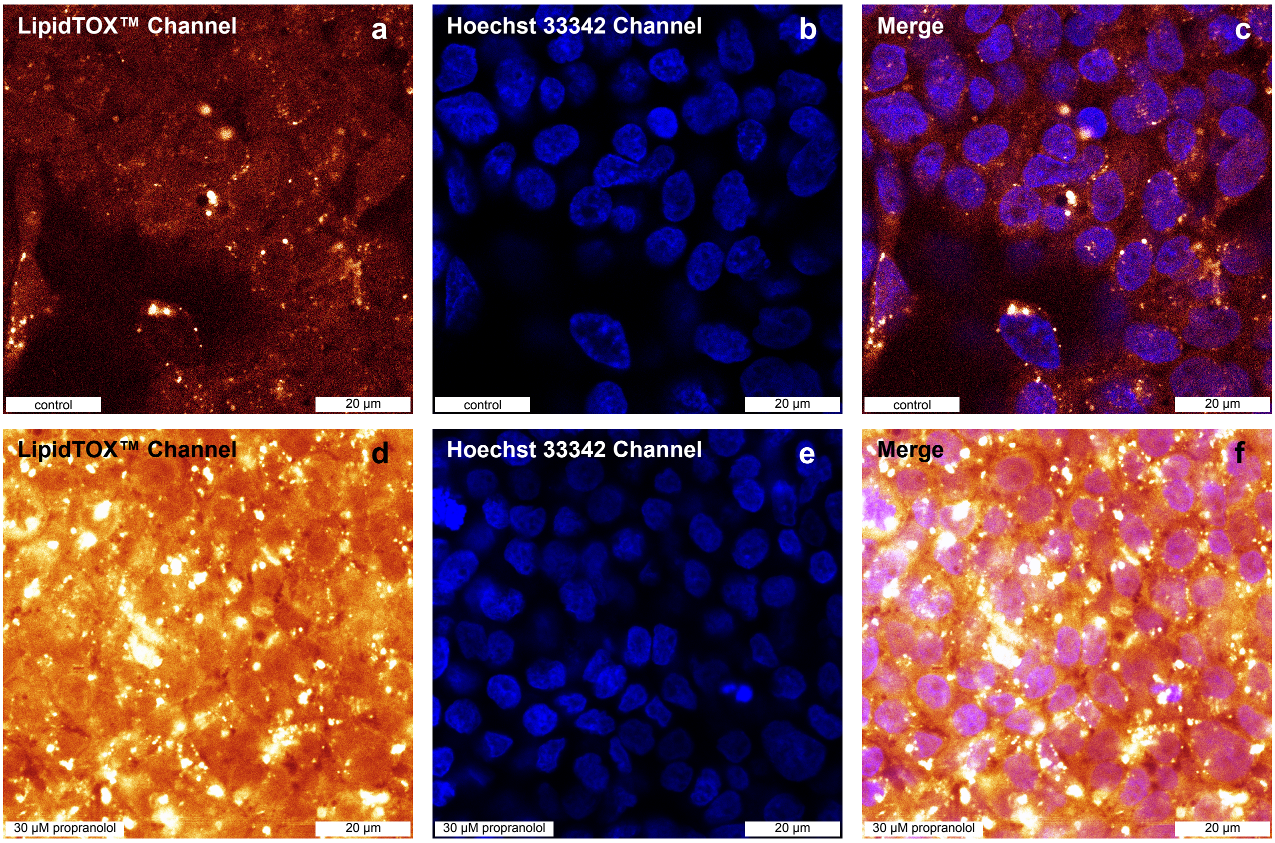

Fig. S9. Phospholipidosis detection using LipidTOX™ in Hep G2 cells. **a** to **c** were obtained without propranolol addition to the medium. **d** to **f** were obtained after exposure for 48 h to medium containing 30 µM propranolol. **a** and **d** were exposed to LipidTOX™ reagent (red fluorescent) and imaged using a 595 nm excitation filter and a 615 emission filter. **b** and **e** were exposed to Hoechst 33342 (blue fluorescent) to stain cell nuclei and imaged with a 352 nm excitation filter and a 461 nm emission filter. **c** and **f** are the merged images corresponding to **a**/**b** and **d**/**e** respectively.

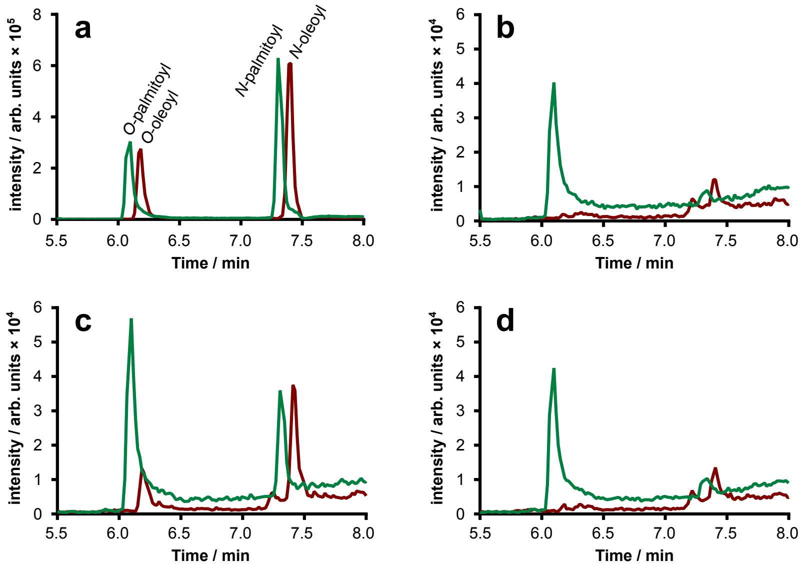

Fig. S10. Solvent extraction tests on acyl propranolol lipid mixtures. *O*- and *N*-palmitoyl propranolol are shown in green; *O*- and *N*-oleoyl propranolol are shown in red. **a**, EICs obtained from an aged POPC/propranolol sample. **b** to **d** show EICs from the solvent fraction after extraction of the sample in **a** with $CHCl_3$ (**b**), $CHCl_3$:MeOH (2:1, **c**) or methyl *tert*-butyl ether (**d**). Chromatograms were obtained using Gradient 2 (methods section).

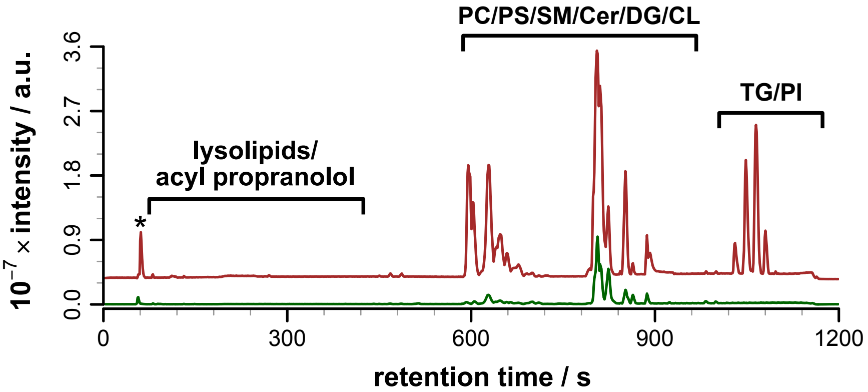

Fig. S11. Base peak normalised LC-MS chromatogram of lipids extracted from Hep G2 cells cultured in a medium containing propranolol (30 µM). Parameters are the same as Fig. S7.

Table S8. Phospholipid and lysolipid species identified within $CHCl_3$/MeOH extracts from Hep G2 cells.

Observed *m/z*	Theoretical *m/z*	$[M+H]^+$ Error (ppm)	Assignment [a]
454.2918	454.2928	2.2	LPE(16:0)
482.3234	482.3241	1.5	LPE(18:0)
502.2926	502.2928	0.3	LPE(20:4)
680.4845	680.4861	2.4	PE(30:0(OH))
708.4623	708.4599	3.4	PE(34:6)
722.5305	722.5331	3.6	PC(30:0(OH))
728.4844	728.4861	2.3	PE(34:4(OH))
728.5236	728.5225	1.5	PC(32:3)
732.5518	732.5538	2.7	PC(32:1)
736.5463	736.5487	3.3	PE(34:0(OH))
738.5084	738.5069	2.0	PE(36:5)
746.6082	746.6058	3.2	PC(34:1)†
748.5247	748.5276	3.9	PE(38:6)‡
754.5399	754.5382	2.3	PC(34:4)
760.4916	760.4912	0.5	PE(38:8)
772.4771	772.4770	0.1	PS(34:3(OH))
776.6183	776.6164	2.4	PE(38:0)
784.5821	784.5851	3.8	PC(36:3)
790.5396	790.5382	1.8	PE(40:7)
794.5720	794.5695	3.1	PE(40:5)
806.5726	806.5695	3.8	PC(38:6)
810.5984	810.6008	2.9	PC(38:4)
813.5496	813.5488	1.0	PI(32:0(OH))†
822.5671	822.5654	2.1	PS(40:5)†
832.6467	832.6437	3.6	PS(40:0)†
834.6035	834.6008	3.2	PC(40:6)
837.5468	837.5488	2.4	PI(34:1)
853.6168	853.6165	0.4	PI(36:0)†
855.4987	855.5018	3.6	PI(36:6)
860.6177	860.6164	1.5	PC(42:7)
860.6713	860.6739	3.0	PC(40:1(OH))
860.6757	860.6750	0.8	PS(42:0)†
863.5659	863.5644	1.7	PI(36:2)
881.5176	881.5175	0.1	PI(38:7)
885.5453	885.5488	4.0	PI(38:5)
887.5651	887.5644	0.8	PI(38:4)
889.5801	889.5801	0.0	PI(38:3)
911.5610	911.5644	3.7	PI(40:6)
929.5779	929.5750	3.1	PI(40:5(OH))
931.5313	931.5331	1.9	PI(42:10)
951.5598	951.5593	0.5	PI(42:8(OH))
959.5664	959.5644	2.1	PI(44:10)

[a] † indicates the presence of alkyl ether substituent; ‡ indicates the presence of alkenyl ether (plasmalogen) substituent.

Table S9. Lipids with significant differences in intensity between Hep G2 cells incubated with propranolol (30 µM) in the medium, and those incubated without propranolol. Analysis was conducted using the xcms package[1] in R[2].

m/z obs	Lipid [a,b]	Formula	Ion	Error (ppm) [b]	r.t. / s	Fold change [c]
754.67474	DG(P-46:6)	$C_{49}H_{88}NO_4$	$[M+NH_4]^+$	5.3	972.8	10
790.5785419	PC(O-38:6)	$C_{46}H_{81}NO_7P$	$[M-H]^-$	3.7	835.0	8
	PC(P-38:5)	$C_{46}H_{81}NO_7P$	$[M-H]^-$	3.7	835.0	8
1011.736606	PI(O-48:4)	$C_{57}H_{104}O_{12}P$	$[M-H]^-$	9.4	956.9	8
	PI(P-48:3)	$C_{57}H_{104}O_{12}P$	$[M-H]^-$	9.4	956.9	8
	PG(50:4)	$C_{57}H_{104}O_{12}P$	$[M+Form]^-$	9.4	956.9	8
720.59021	PC(O-32:0)	$C_{40}H_{83}NO_7P$	$[M+H]^+$	0	832.3	7
	CerP(t40:0)	$C_{40}H_{83}NO_7P$	$[M+H]^+$	0	832.3	7
802.4956973	PS(34:0)	$C_{40}H_{78}NO_{10}PK$	$[M+K]^+$	4.7	595.8	-7
773.5218128	MGDG(36:6)	$C_{45}H_{73}O_{10}$	$[M-H]^-$	1.2	589.3	6
1037.750393	PI(O-50:5)	$C_{59}H_{106}O_{12}P$	$[M-H]^-$	7.3	956.9	6
	PI(P-50:4)	$C_{59}H_{106}O_{12}P$	$[M-H]^-$	7.3	956.9	6
797.5334592	PG(38:5)	$C_{44}H_{78}O_{10}P$	$[M+H]^+$	1.0	599.6	5
878.81976	TG(52:1)	$C_{55}H_{108}NO_6$	$[M+NH_4]^+$	3.1	1080.5	-2
904.8373	TG(54:2)	$C_{57}H_{110}NO_6$	$[M+NH_4]^+$	5.0	1082.1	-2
820.7410	TG(48:2)	$C_{51}H_{98}NO_6$	$[M+NH_4]^+$	2.6	1029.6	-2
848.7718	TG(50:2)	$C_{53}H_{102}NO_6$	$[M+NH_4]^+$	1.9	1063.2	-2

[a] Key: CerP, ceramide phosphate; DG, diacylglycerol; PA, phosphatidic acid; PC, phosphatidylcholine; PE, phosphatidylethanolamine; PG, phosphatidylglycerol; PI, phosphatidylinositol; PS, phosphatidylserine; TG, triacylglycerol. The 'O-' and 'P-' prefixes refer to *O*-alkyl and *O*-alkenyl (plasmenyl) ethers respectively. Matches shown are the most likely on the basis of either a low mass error or likelihood based on the acyl carbon count.

[b] matched against the LIPD MAPS® database, http://www.lipidmaps.org.

[c] negative values (in red) correspond to a decrease in the presence of propranolol.

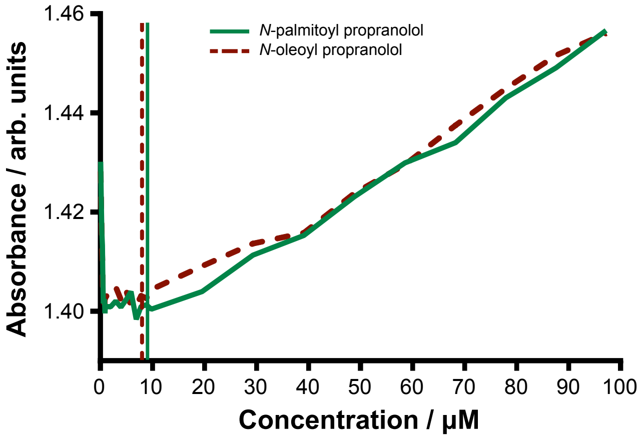

Fig. S12. Rhodamine 6G absorbance in the presence of increasing concentrations of *N*-palmitoyl propranolol (solid green line) and *N*-oleoyl propranolol (red dashed line). The corresponding vertical line indicates the critical micelle concentration for each species (10 µM and 9 µm respectively for *N*-palmitoyl and *N*-oleoyl propranolol). Similar analysis for OPC and PPC gave respective CMC values of 7 µM and 4 µM, as expected based on literature precedents[9–11].

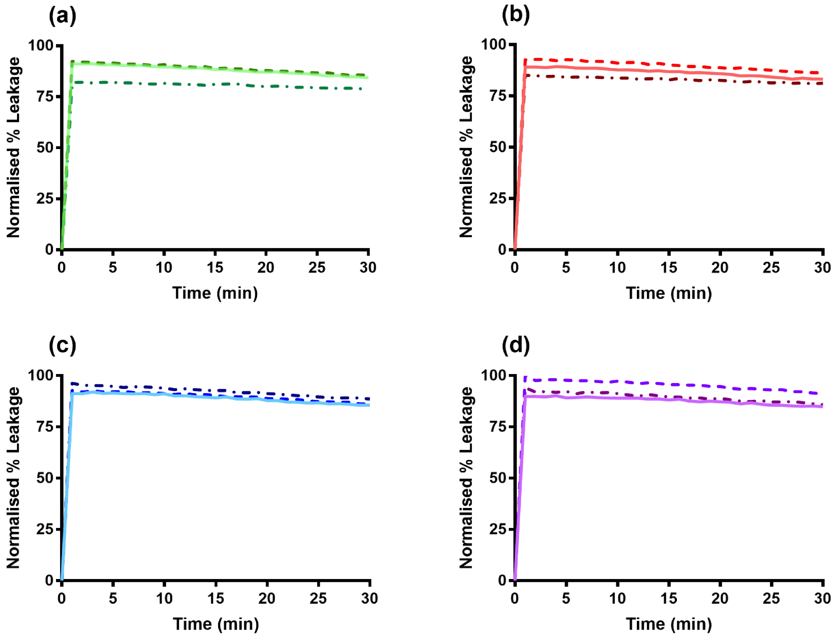

Fig. S13. Fluorescence emission intensity at 530 nm (λ_{ex} 360 nm) from liposomes loaded with 8-aminonaphthalene-1,3,6-trisulfonic acid (ANTS, 12.5 mM) and *p*-xylene-bis-pyridinium bromide (DPX, 45 mM) at pH 7.4 and 37 °C following treatment with 1/2-palmitoyl-*sn*-glycero-3-phosphocholine (PPC; **a**), 1/2-oleoyl-*sn*-glycero-3-phosphocholine (OPC; **b**), *N*-palmitoyl propranolol (**c**) and *N*-oleoyl propranolol (**d**). All plots are normalised with respect to the intensity following treatment of the liposomes with Triton X-100 (100% release). Solid, dashed and dotted/dashed lines correspond respectively to 1, 5 and 100 mol% of additive with respect to lipid.

References

1. C. A. Smith, E. J. Want, G. O'Maille, R. Abagyan and G. Siuzdak, *Anal. Chem.*, 2006, **78**, 779–787.
2. R Core Team, 2017. R: A Language And Environment For Statistical Computing.
3. J. R. Masters and G. N. Stacey, *Nat. Protoc.*, 2007, **2**, 2276–2284.
4. R. Hamid, Y. Rotshteyn, L. Rabadi, R. Parikh and P. Bullock, *Toxicol. in Vitro*, 2004, **18**, 703–710.
5. P. P. Bonsen, G. H. de Haas, W. A. Pieterson and L. L. van Deenen, *Biochim. Biophys. Acta*, 1972, **270**, 364–382.
6. P. Becher, *J. Phys. Chem.*, 1962, **66**, 374–375.
7. A. M. Kawamoto and M. Wills, *J. Chem. Soc., Perkin Trans. 1*, 2001, 1916–1928.
8. G. F. White, K. I. Racher, A. Lipski, F. R. Hallett and J. M. Wood, *Biochim. Biophys. Acta*, 2000, **1468**, 175–186.
9. P. O. Eriksson, G. Lindblom and G. Arvidson, *J. Phys. Chem.*, 1987, **91**, 846–853.
10. R. E. Stafford, T. Fanni and E. A. Dennis, *Biochemistry*, 1989, **28**, 5113–5120.
11. L.-J. Chen, S.-Y. Lin and C.-C. Huang, *J. Phys. Chem. B*, 1998, **102**, 4350–4356.
